# Nucleophilic Thiols Reductively Cleave Ether Linkages in Lignin Model Polymers and Lignin

**DOI:** 10.1002/cssc.202001238

**Published:** 2020-08-07

**Authors:** Grace E. Klinger, Yuting Zhou, Juliet A. Foote, Abby M. Wester, Yanbin Cui, Manar Alherech, Shannon S. Stahl, James E. Jackson, Eric L. Hegg

**Affiliations:** ^1^ Department of Chemistry Michigan State University East Lansing, MI 48824 USA; ^2^ Department of Biochemistry & Molecular Biology Michigan State University East Lansing, MI 48824 USA; ^3^ DOE Great Lakes Bioenergy Research Center Michigan State University East Lansing, MI 48824 USA; ^4^ DOE Great Lakes Bioenergy Research Center University of Wisconsin-Madison Madison WI 53706 USA; ^5^ Department of Chemistry University of Wisconsin-Madison Madison WI 53706 USA

**Keywords:** ether, homogeneous catalysis, lignin, polymers, sulfur

## Abstract

Lignin may serve as a renewable feedstock for the production of chemicals and fuels if mild, scalable processes for its depolymerization can be devised. The use of small organic thiols represents a bioinspired strategy to cleave the β‐O‐4 bond, the most common linkage in lignin. In the present study, synthetic β‐O‐4 linked polymers were treated with organic thiols, yielding up to 90 % cleaved monomer products. Lignin extracted from poplar was also treated with organic thiols resulting in molecular weight reductions as high as 65 % (*M*_n_) in oxidized lignin. Thiol‐based cleavage of other lignin linkages was also explored in small‐molecule model systems to uncover additional potential pathways by which thiols might depolymerize lignin. The success of thiol‐mediated cleavage on model dimers, polymers, and biomass‐derived lignin illustrates the potential utility of small redox‐active molecules to penetrate complex polymer matrices for depolymerization and subsequent valorization of lignin into fuels and chemicals.

## Graphical abstract

**Thiols to the rescue**: Thiols cleave ether bonds in polymeric oxidized lignin by penetrating the polymeric network of lignin and cleaving ether bonds activated by neighboring carbonyls.

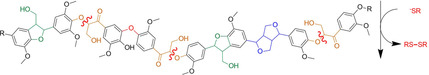

The need to replace fossil fuels with renewable feedstocks for the manufacture of chemicals and fuels is critical due to finite petroleum resources and rising greenhouse gas emissions.^1^ Most efforts to make transportation fuels and chemicals from lignocellulosic biomass have focused on depolymerization of cellulose and fermentation of the resulting sugars to alcohols.^2^ However, the carbon‐rich lignin fraction could also displace substantial fossil petroleum if practical methods were available for its depolymerization into chemically tractable fragments.^2^

Lignin is an energy‐ and carbon‐rich aromatic polymer found in plant cell walls. Historically treated as a waste byproduct of the pulp and paper industry, it has recently gained attention as a possible bio‐based substitute for petroleum feedstocks in fuel and chemical production.^2^ Lignin depolymerization, however, is energy intensive and costly due to the chemical recalcitrance of the linkages between the aromatic propylphenol subunits. Additionally, simple oxidative cleavage methods decrease the energy density of the products and often lead to the formation of new undesired covalent crosslinks.

A number of non‐oxidative lignin depolymerization methods have been developed, many of which focus on the cleavage of the aryl ethers in β‐O‐4 bonds, the most prevalent linkages in lignin. Reductive methods for ether cleavage often begin with a nucleophilic halide paired with a Lewis acid.^3^ Derivatization followed by reductive cleavage (DFRC) has been developed as a lignin analysis procedure, cleaving ether bonds via treatment with acetyl bromide and subsequent reduction with zinc metal.^4^ In the acidolysis method, hydrochloric acid, heat, and dioxane are used to cleave aryl ethers,^5^ but low cleavage yields led researchers to develop an improved variation called thioacidolysis. This acidic solvolysis uses boron trifluoride etherate, a potent Lewis acid, together with ethanethiol in dioxane to cleave aryl ether bonds. The resulting thiolated monomers can then be reduced with Raney nickel.^6^ Like DFRC, thioacidolysis is mainly used to analyze lignin.

Catalytic hydrogenolysis, another reductive lignin cleavage technique, utilizes pressurized hydrogen and heat in the presence of catalysts such as Raney® nickel,^7^, ^8^ ruthenium, platinum, or palladium.^8^, ^9^ Condensation of the lignin fragments after depolymerization, a common problem of hydrogenolysis and other high temperature lignin depolymerization reactions, has been minimized by Luterbacher and co‐workers by using aldehydes as stabilizers.^10^ The above metal catalysts are also key in electrocatalytic hydrogenation (ECH), a reductive technique in which lignin linkages are cleaved in an electrochemical cell.^11^

Oxidation of the C_α_−OH in β‐O‐4 lignin models, followed by reductive cleavage, an overall redox‐neutral process, has met with some success. Common oxidants such as (2,2,6,6‐tetramethylpiperidin‐1‐yl)oxyl (TEMPO^+^) or 2,3‐dichloro‐5,6‐dicyano‐1,4‐benzoquinone (DDQ) readily oxidize the HC_α_−OH to a C_α_=O carbonyl. This lowers the C−O ether bond dissociation energy, allowing reductants such as zinc used by Westwood and co‐workers,^12^ or photo‐reductive iridium complexes used by Stephenson and co‐workers,^13^ to cleave these α‐oxidized model β‐O‐4 dimers. Another effective redox neutral process for the cleavage of α‐oxidized β‐O‐4 linkages uses formic acid and water, and has successfully been applied to lignin from various sources.^14^ Despite these advances, new simple and inexpensive processes remain of interest to diversify lignin depolymerization strategies.

Thiols are both strong nucleophiles and reductants, and they have been employed in applications ranging from protein denaturation to thiol‐click chemistry.^15^ Thiols also play key roles in biochemical pathways, including one that cleaves ether bonds vicinal to carbonyl groups in α‐oxidized lignin (Figure 1a). A relatively unexplored avenue for ether cleavage is a biomimetic approach, mimicking etherases such as the glutathione‐dependent enzymes from wood‐digesting bacteria like *Sphingobium* sp strain SYK‐6.^16^ Glutathione (GSH) is a tripeptide used by the Lig enzymes E, P, F, and BaeAB (etherases), and Lig enzyme G and GST_Nu_ (lyase).^16^, ^17^ This cofactor contains a cysteine (thiol) residue that performs a protein‐assisted S_N_2 reaction on an α‐keto lignin dimer, forming a thioether. The thioether is then reduced by another glutathione resulting in the formation of a disulfide bond and liberation of an enolate that is protonated to form the ketone.^16^ The net result is addition of two hydrogen atoms across the β‐O‐4′‐aryl ether C_β_−O bond, reductively fragmenting the α‐keto β‐O‐4 linkage, as summarized in Figure 1a.

**Figure 1:**
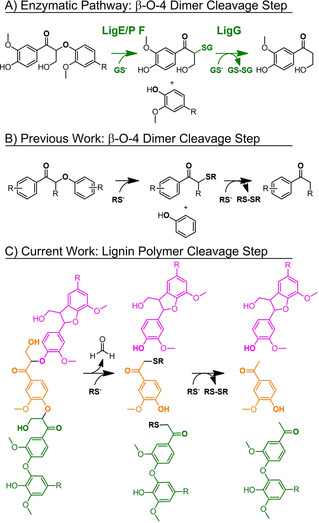
β‐aryl ether cleavage via thiol nucleophilic attack. A) Enzymatic β‐aryl ether cleavage of C_α_‐oxidized lignin dimers with glutathione (GSH). B) Our previous work, focusing on β‐O‐4 model dimers cleavage with organic thiols (RSH). C) This work, focusing on lignin and lignin‐like polymer cleavage using organic thiols to form lignin fragments that can be fed into other processes for subsequent valorization.

Inspired by the chemistry harnessed by *Sphingobium's* Lig enzymes to achieve reductive depolymerization of lignin dimers, we recently reported that organic thiols, under alkaline polar aprotic solvent conditions, can cleave keto aryl ether bonds in model lignin systems with nearly 100 % yields (Figure 1b).^18^ In this study, we examined the nucleophilic and reductive capabilities of free organic thiols to cleave ether bonds in polymeric lignin (Figure 1c). The ability of small, diffusible redox mediators to penetrate and chemically attack the lignin network is a potential advantage relative to the Lig enzymes which are mostly limited to small lignin fragments.^17^, ^19^ Furthermore, these diffusible thiol‐based redox carriers can theoretically be recycled using 2‐electron/2‐proton reduction of the disulfide product in an electrochemical cell (Figure S28), analogous to the electrochemical oxidative mediators employed by Stahl and co‐workers.^20^ Herein, we first explore alkaline thiol‐mediated cleavage of model β‐aryl ether polymers into monomeric products, and then extend our studies to lignin extracted from poplar, and to other lignin‐type linkages.

The ability of small organic thiols to penetrate polymeric networks should enable nucleophilic cleavage of lignin from cellulosic biomass. To test thiols’ ability to access cleavage sites in bulky polymeric systems, three synthetic β‐O‐4‐linked polymers were constructed. Degrees of polymerization were approximately 10, as analyzed through elemental analysis (Supporting Information, page S6). These insoluble^21^ 10‐mers were subjected to depolymerization using neat 1,3‐propanedithiol as shown in Figure 2. The dithiol,1,3‐propanedithiol, was chosen for its reducing abilities due to stabilization of the oxidized five‐membered ring product through an intramolecular disulfide bond. Synthetic polymer A (poly‐4‐hydroxyacetophenone), a polyphenol made entirely of β‐O‐4 linkages, was depolymerized resulting in soluble monomer yields as high as 90 %. Synthetic polymer B (poly‐vanillone), a methoxylated analog of polymer A, was also cleaved with up to 80 % yields. This lower yield may be due either to the electronic effects of the methoxy groups that decrease the partial positive charge on the β‐position or to steric effects as observed previously in our β‐O‐4 model studies.^18^ Reaction of Polymers A and B with 2‐mercaptoethanol (BME) was slightly less successful than 1,3‐propanedithiol but still resulted in monomer yields of up to 70 % (Figure S6). This trend was also seen in previous work^18^ on dimer cleavage. The low sulfur content, <2 %, of the remaining polymer (Figure S7) indicates that the thiol‐dependent depolymerization reaction completed both the S_N_2 ether cleavage and the reductive elimination of the resulting thioether, resulting in disulfide formation and release of the ketone fragment. Cleavage of the β‐hydroxymethylated analog of polymer B, a more representative model of β‐O‐4 linkages in lignin, would be expected to yield the same products as those from polymer B due to loss of the hydroxymethyl moiety on the β‐position via the retro‐aldol side reaction seen in our previous work.^18^ This process would form polymer B, which would be cleaved as seen above. Importantly, synthetic polymer C (reduced poly‐4‐hydroxyacetophenone), the α‐hydroxy analogue of polymer A, was unreactive to thiol mediated depolymerization, as expected. As seen both in our earlier work^18^ and in the enzymatic pathway being mimicked,^22^ oxidation of the α‐position is required for nucleophilic attack by the thiol.

**Figure 2:**
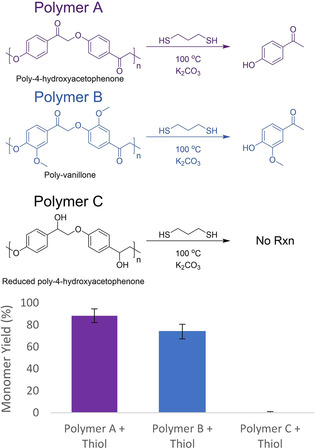
Top: Synthetic model β‐O‐4 polymer cleavage reaction (3 h) with 1,3‐propanedithiol to yield monomer cleavage products. A) poly‐4‐hydroxyacetophenone synthetic polymer, B) poly‐vanillone synthetic polymer, C) reduced poly‐4‐hydroxyacetophenone. Bottom: Yields of monomers cleaved from top reactions. No cleavage products were seen from polymer C. Yields reported are incremental monomer yields in excess of those obtained from reactions performed without thiol.

With the above promising results on lignin polymer model systems, efforts were extended to an exploration on biomass‐derived lignin as represented by Figure S8. All lignin used in this study, unless otherwise stated, was isolated via the copper‐catalyzed alkaline hydrogen peroxide (Cu‐AHP) pretreatment of poplar.^23^ This lignin is slightly oxidized at the α position.^24^ Lignin reaction conditions were optimized, testing various solvents (Figures S9 and S10), reaction times (Figure S11), thiols (Figures S15–S17), and work‐up procedures (Figures S12–S14 and Figure S24) to optimize conditions for depolymerization and analysis. The largest molecular weight decreases were seen using neat thiol for 24 h (Figure S11), with dithiothreitol (DTT) and BME as the most successful thiols (Figure S10).

With the above reaction parameters optimized, Cu‐AHP lignin^23^ was stirred in the presence of powdered K_2_CO_3_ with either neat BME (Figures 3a and S15), neat DTT (Figure S15), or neat 1,3‐propanedithiol (Figures S23a,c) at 100 °C for 24 h. For molecular weight analysis, mixtures were diluted with water to solubilize the base and lignin (see Supporting Information, page S7). Large decreases in molecular weight were observed when Cu‐AHP lignin was depolymerized with BME (Figure 3a, c) and DTT (Figures S15, S16) despite these thiols being less successful in dimer and synthetic oligomer cleavage. This cleavage resulted in 60–80 % polymer mass loss, presumably through formation of small fragments that are acid soluble (Figure S18). Analysis of the −OH content showed a doubling of guaiacyl −OH sites, confirming that ether cleavage had occurred (Figure S19). Similarly, in the remaining lignin, the oxidized β‐O‐4 dimer NMR signal decreased after treatment with DTT and BME, indicating that these oxidized fragments had been removed from the polymer (Figure S20). Finally, lignin analysis via thioacidolysis, a lignin analysis technique to quantify β‐O‐4 content, also showed that the β‐O‐4 content decreased, presumably due to the thiolate‐mediated ether cleavage (Figures S21 and S22).

**Figure 3:**
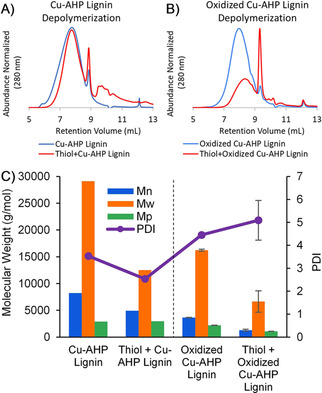
Gel permeation chromatograms of A) Cu‐AHP lignin before and after treatment with BME, B) Cu‐AHP lignin oxidized with Bobbitt's salt before and after treatment with BME. C) Molecular weight distributions for number average molecular weight (*M*_n_) in blue, weight average molecular weight (*M*_w_) in orange, highest‐peak molecular weight (*M*_p_) in green, and polydispersity (PDI) in purple. Upon BME treatment, larger molecular weight decreases were observed for the oxidized Cu‐AHP lignin than for the unoxidized Cu‐AHP lignin. As a control, non‐thiol‐treated lignin was prepared in the same way as thiol‐treated lignin (water and base) to account for any base‐mediated cleavage or condensation (see Supporting Information, page S7).

Interestingly, while BME and DTT were moderately successful at Cu‐AHP lignin depolymerization, 1,3‐propanedithiol under neat conditions and over various reaction times (Figure S17) was unsuccessful, with little to no decrease in the Cu‐AHP lignin molecular weight. These results contrast with the high cleavage yields achieved with 1,3‐propanedithiol treatment of model dimers or synthetic oligomers (Figures 2 and S6).

Noting the importance of the α‐keto moiety in activating the cleavage, we explored further oxidation of the lignin with Bobbitt's salt,^25^ which resulted in an approximately 5‐fold increase in oxidation of the α‐OH groups (Figure S23e,f). Incubation of BME with this oxidized Cu‐AHP lignin resulted in the *M*_n_ (number average molecular weight) decreasing by approximately 65 % compared to approximately 40 % for BME treatment of the unoxidized Cu‐AHP lignin (Figure 3). The use of the less reactive thiol, 1,3‐propanedithiol, on the oxidized Cu‐AHP lignin resulted in an *M*_n_ decrease of 49 % (Figure S23b,c). Thus, oxidation enhances thiol‐mediated depolymerization.

Importantly, performing the Cu‐AHP lignin depolymerization reaction on a 1 g scale (Figure S27) proved to be just as efficient as the base case 100 mg scale, indicating that the lignin depolymerization reaction can be successfully scaled up. Furthermore, scale‐up and variations in procedural details of the Cu‐AHP lignin isolation did not alter the lignin properties (e. g., NMR chemical shift, molecular weight, solubility) or affect depolymerization (Figures S25 and S26), demonstrating the robustness of the process. While the exact reaction mechanism remains to be verified, our results clearly demonstrate that diffusible organic thiols are able to penetrate and cleave the polymeric network not only of synthetic linear polymers, but also of oxidized poplar lignin.

The successful depolymerization of lignin, which comprises five other linkages in addition to the β‐O‐4 bond (Figure S8), prompted us to look at some of these other linkages to see if they might also be cleaved using the potent nucleophilicity of small organic thiols and thiolates. The β‐5/α‐O‐4 and the 4‐O‐5 linkages are the two other ether bonds found in lignin. In addition, acetal‐type linkages may occur in oxidized lignins via combinations of aldehyde or ketone and alcohol sites.^10^ To probe for possible cleavage by thiols, we treated model dimers (dimers defined here as two aromatic rings linked through one of the known ether lignin linkages) under the optimized conditions^18^ of BME in refluxing acetonitrile with K_2_CO_3_ (Figure 4).

**Figure 4:**
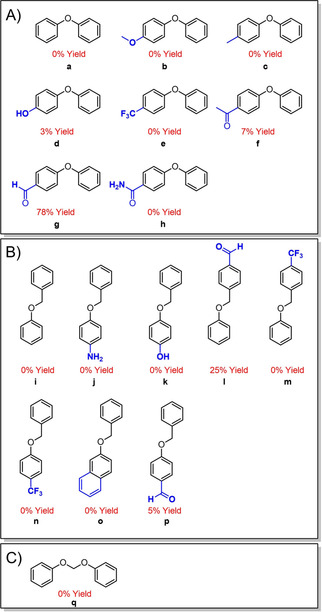
Non‐β‐O‐4 model lignin dimers studied for monomer product formation using thiols: A) diaryl ethers modelling the 4‐O‐5 linkage, B) benzyl phenyl ethers modelling the α‐O‐4/β‐5, and C) diphenoxymethane, an acetal. Yields of phenolic monomer are indicated in red.

In the case of the 4‐O‐5 linkage, simple diaryl ethers were used as models. Consistent with leaving group ability and previous Hammett studies, only the *p*‐ketone and *p*‐aldehyde‐substituted diaryl ethers were cleaved (Figures 4A and S1; Table S1) with yields of 7 % and 78 %, respectively. These reactions presumably proceed via a nucleophilic aromatic substitution mechanism (Figure S5), yielding the easily detected free phenol product. Other diaryl ethers with *p*‐substituted electron withdrawing or donating groups were unreactive to thiol‐mediated cleavage. Thus, thiolate‐based cleavage of 4‐O‐5 ether linkages in lignin appear unlikely, unless prior pretreatment methods produce activating carbonyl moieties.

To test the reactivity of β‐5/α‐O‐4 linkages, simple benzyl phenyl ethers were used as model dimers. Like the 4‐O‐5 diaryl ether dimers, the β‐5/α‐O‐4 model dimers were generally unreactive. Again, carbonyl moieties enabled a small amount of reaction (Figure 4B; Figure S3; Table S2); only substrates with *p*‐aldehyde groups either on the benzyl or on the phenoxyl sides of the dimer released the phenolic products upon thiol treatment, doing so in 25 % and 5 % yields, respectively.

Despite representing an unhindered primary site for potential S_N_2 attack, with two phenoxide leaving groups, the acetal diphenoxymethane, with no carbonyls to activate it, was unreactive to cleavage by thiol (Figure 4C; Figure S2).

Both the 4‐O‐5 and β‐5/α‐O‐4 model dimer cleavage reactions are evidently conventional nucleophilic displacements; addition of butylated hydroxytoluene (BHT, 1 equiv.), a known radical scavenger, did not affect the yields of cleavage in either substrate class (Figure S4). Thus, as seen in our previous work with the β‐O‐4 linkage,^18^ we conclude that these reactions do not proceed via free radicals. These two other cleavage processes may contribute a small amount of additional thiol‐mediated depolymerization in lignin that is partially or fully α‐oxidized. Therefore, the oxidation that activates the β‐O‐4 cleavage via α‐carbonyl formation may also enable additional ether cleavages for a more fully depolymerized lignin.

In summary, building on our prior model‐based studies, we have now demonstrated that small, diffusible thiols can penetrate complex matrices, access the polymer backbone, and act as redox mediators to reductively cleave ether linkages in polymeric substrates, including biomass‐derived lignin, that bear the α‐keto functionality. Small organic thiols can readily cleave simple model lignin oligomers containing α‐keto β‐O‐4 linkages with near complete mass balance. The penetration and depolymerization of natural oxidized lignin suggest that this strategy could be a viable technique for lignin fragmentation for further downstream valorization. Furthermore, the successful, albeit modest, cleavage of non‐β‐O‐4 linkages potentially expands the utility of this thiol‐mediated depolymerization process. These findings open the door for future studies in which electrochemical 2‐electron/2‐proton reductions of the disulfide byproducts would regenerate the thiols, enabling net electrocatalytic lignin depolymerization.

## Experimental Section

**GPC analysis of lignin depolymerization**: For molecular weight analysis, the thiol‐treated lignin mixture (100 mg of lignin, 1 mL of thiol, and 100 mg of K_2_CO_3_) was cooled to room temp, dissolved in 20 mL of water, centrifuged, and injected directly to the HPLC‐GPC using the conditions stated in the Supporting Information. Non‐thiol‐treated lignin was worked‐up in the same manner (100 mg of lignin dissolved in 20 mL of water with 100 mg of K_2_CO_3_) and injected directly to the HPLC as a control comparison. Retention times were compared to a sodium polystyrene sulfonate kit from Scientific Polymer Products (*M*_n_: 1440–85 600 g mol^−1^).

**NMR analysis of lignin**: Thiol‐treated lignin was solubilized in 20 mL of water and precipitated with H_2_SO_4_ to pH 2, centrifuged, washed with acidic water, frozen, and lyophilized. The resulting dry depolymerized lignin was then characterized by NMR spectroscopy. Each lignin depolymerization reaction was accompanied by a control reaction with the corresponding non‐thiol‐treated lignin worked‐up in the same manner.

**Synthetic polymer depolymerization**: Synthetic polymer (10 mg), dried powdered K_2_CO_3_ (100 mg), and thiol (0.5 mL) were added to a glass vial (≈5 mL) and closed with an aluminum‐lined cap. The mixture was stirred at 100 °C in an oil bath for 3 h. The solution was dissolved in 20 mL of water and analyzed by LCMS according to the specifications provided in the General Information section in the Supporting Information. For sulfur analysis, the reaction was scaled up 5x and the depolymerized thiol‐treated solution was centrifuged, decanted, and the remaining solid was washed 3x with alkaline water. This washed solid was frozen, lyophilized, and the dried powder was sent to A&L Great Lakes (Fort Wayne, IN) for sulfur analysis.

**Lignin depolymerization**: Thiol‐treated lignin was treated and processed as follows unless otherwise noted: Lignin (100 mg) and dried powdered K_2_CO_3_ (100 mg) were added to a glass vial (≈10 mL) with a stir bar, capped with a septum, and purged with nitrogen. Thiol (1 mL) was added to the reaction, which was then capped with an aluminum‐lined cap and stirred at 100 °C in an oil bath for 1–24 h. The aqueous solubilized thiol‐treated lignin was precipitated by acidification with H_2_SO_4_ (HCl for sulfur analysis) to pH 2, centrifuged, washed with acidic water, frozen, and lyophilized. The resulting dry depolymerized lignin was then characterized by the following: elemental analysis, mass balance, thioacidolysis, and NMR spectroscopy.

**Dimer cleavage**: Lignin model dimers (10 mg) and dried powdered K_2_CO_3_ (≈100 mg) were added to an oven‐dried round‐bottom flask (50 mL) equipped with a stir bar, condenser, septum, and nitrogen‐filled balloon, and the entire apparatus was purged with N_2_. Acetonitrile (20 mL) and thiol (10 equiv. relative to dimer) were added through a septum, and the reaction was refluxed with periodic sampling for HPLC analysis to monitor progress over 24 h.

## Supporting information

As a service to our authors and readers, this journal provides supporting information supplied by the authors. Such materials are peer reviewed and may be re‐organized for online delivery, but are not copy‐edited or typeset. Technical support issues arising from supporting information (other than missing files) should be addressed to the authors.

Supplementary
