# Supplementary material for: Nucleophilic Thiols Reductively Cleave Ether Linkages in Lignin Model Polymers and Lignin
Source: ChemSusChem. 2020 Aug 7;13(17):4394–9. doi: 10.1002/cssc.202001238 (PMC7540407; doi:10.1002/cssc.202001238)
Supplement: Supplementary file 1 — Supplementary [file CSSC-13-4394-s001.pdf]

ChemSusChem

Supporting Information

Nucleophilic Thiols Reductively Cleave Ether Linkages in Lignin Model Polymers and Lignin

Grace E. Klinger, Yuting Zhou, Juliet A. Foote, Abby M. Wester, Yanbin Cui, Manar Alherech, Shannon S. Stahl, James E. Jackson,* and Eric L. Hegg*© 2020 The Authors. Published by Wiley-VCH GmbH.
This is an open access article under the terms of the Creative Commons Attribution License, which permits use, distribution and reproduction in any medium, provided the original work is properly cited.This publication is part of a Special Issue focusing on "Lignin Valorization: From Theory to Practice". Please visit the issue at .

Author Contributions

G.K. Conceptualization:Equal; Formal analysis:Lead; Investigation:Lead; Methodology:Lead; Writing - Original Draft:Lead; Writing - Review & Editing:Equal
Y.Z. Investigation:Supporting
J.F. Investigation:Supporting
A.W. Investigation:Supporting
Y.C. Investigation:Supporting; Writing - Review & Editing:Supporting
M.A. Investigation:Supporting; Writing - Review & Editing:Supporting
S.S. Supervision:Supporting; Writing - Review & Editing:Supporting
J.J. Conceptualization:Equal; Project administration:Equal; Supervision:Equal; Writing - Review & Editing:Equal
E.H. Conceptualization:Equal; Funding acquisition:Lead; Project administration:Equal; Supervision:Equal; Writing - Review & Editing:Equal

Supporting Information
©Wiley-VCH 2016
69451 Weinheim, Germany

Nucleophilic Thiols Reductively Cleave Ether Linkages in Lignin Model Polymers and Lignin

Grace E. Klinger, Yuting Zhou, Juliet A. Foote, Abby M. Wester, Yanbin Cui, Manar Alherech, Shannon, S. Stahl, James E. Jackson, and Eric L. Hegg**

Contents

I. GENERAL INFORMATION S3

II. SYNTHESIS OF MODEL LIGNIN DIMERS AND OLIGOMERS S4

III. GENERAL PROCEDURE FOR CLEAVAGE REACTIONS AND ANALYSIS S7

- I. DIMERS S7
- II. SYNTHETIC POLYMER S7
- III. LIGNIN S7

IV. CLEAVAGE OF 4-O-5 MODELS S10

V. CLEAVAGE OF A-O-4/B-5 MODELS S13

VI. DIMER CONTROL EXPERIMENTS S15

VII. PROPOSED DIMER CLEAVAGE MECHANISM S17

VIII. SYNTHETIC POLYMER CLEAVAGE S18

IX. LIGNIN POLYMER MODEL S21

X. LIGNIN CLEAVAGE S22

XI. CLEAVAGE OF OXIDIZED LIGNIN S40

XII. LIGNIN CONTROL EXPERIMENTS S43

XIII. PROPOSED ELECTROCHEMICAL CYCLE FOR THE THIOL-MEDIATED OXIDIZED B-O-4 CLEAVAGE S49

XIV. REFERENCES S50

I. General Information

Chemicals were tested for purity using ^{1}H NMR prior to use. Benzyl phenyl ether, 4-benzyloxyaniline hydrochloride 98%, 4-(benzyloxy)phenol, 4-(phenoxymethyl)benzaldehyde, 4-(benzyloxy)benzaldehyde 98%, 4-benzyloxybenzotriflouride 95%, 4-(benzyloxy)benzonitrile, benzyl 2-naphthyl ether, diphenyl ether, 4-phenoxyphenol, 4'-phenoxyacetophenone, diphenoxymethane 98%, acetonitrile 99.5+%, 1,3-propanedithiol 99%, 2-mercaptoethanol, 2-chloro-4,4,5,5-tetramethyl 1,3,2-dioxaphospholane were purchased from Sigma Aldrich (St. Louis, MO), TCI Ltd, or Alfa Aesar (Haverhill, MA). DL-dithiothreitol > 99% was purchased from Gold Biotechnology (Olivette, MO). All water used was filtered with a Millipore SAS Milli-Q® Reference Water Purification system. All reactions were performed under a nitrogen atmosphere using a balloon unless otherwise specified. Column purification was accomplished using Silicycle SiliaFlash P60 silica gel (40-63 µm). Thin layer chromatography (TLC) was performed on aluminum silica gel 60 F-254 plates and the bands were visualized using short wave UV light (254 nm). HPLC analysis was performed using an Agilent 1260 Infinity equipped with a G1315D 1260 diode array detector VL, monitoring at 280 nm and recording from 190-400 nm and a G1362 refractive index detector. For dimer and monomer analysis, a Supelco Ascentis Express C18 column 15 cm x 4.6 cm, 2.7 µm was used in isocratic mode at 0.4 mL/min with a mobile phase of 70:30 acetonitrile:water, adjusted to 60:40 for more difficult separations; 5 µL sample injections included phenanthrene (0.28 mM) as an internal standard; external standards were run during each sequence of analysis. Lignin analysis was performed using a Waters Ultrahydrogel 250 7.8 x 300 mm gel permeation chromatography (GPC) column attached to a Waters Ultrahydrogel 6 x 40 mm guard column held at a constant 40 °C temperature in isocratic mode at 0.7 mL/min with a mobile phase of 0.005 M NaOH in 80/20 0.1 M aqueous sodium nitrate/acetonitrile; 25 µL injections included acetophenone (8.6 mM) as an internal standard with polystyrene sulfonic acid external standards run during each sequence. Instrumental control, data acquisition, and data processing for the HPLC were performed with Agilent ChemStation software. LC-MS analyses employed a Waters Xevo G2-XS UPLC/MS/MS equipped with a Quadrupole/Time-of-Flight system (QTOF). ^{1}H and ^{31}P NMR spectra were recorded using Agilent DDR2 500 MHz NMR spectrometers equipped with 7600AS 96 sample autosamplers running VnmrJ 3.2A and referenced to residual solvent peaks. 2D-NMR spectra were recorded using a Varian 600 MHz superconducting NMR-Spectrometer operating at 599.892 MHz interfaced with a Dell Precision T3500, running CentOS 5.6 with VnmrJ 3.2A and a Bruker Avance 900 MHz superconducting NMR-Spectrometer.

II. Synthesis of Model Lignin Dimers and Oligomers

General Protocol for 4-O-5 Synthesis: The following 4-O-5 dimers were prepared using a literature procedure[1] unless otherwise noted. A mixture of aryl bromide (2 mmol), phenol (3 mmol), Cs_2CO_3 (4 mmol), copper iodide (0.2 mmol), *N*,*N*-dimethylglycine hydrochloride salt (0.6 mmol), and dioxane (100 mL) were sealed in a round bottom flask and heated at 90 °C under a nitrogen atmosphere, monitoring for completion by TLC. The reaction was cooled and extracted using EtOAc and water. The organic layer was separated, and the aqueous layer was extracted with additional EtOAc. The combined organic layers were washed with brine, dried over Na_2SO_4, and concentrated *in vacuo*. The residual oil was purified by column chromatography using EtOAc/hexane (1:30) to afford the product.

4-Phenoxybenzonitrile: Spectral data are consistent with those reported in the literature.[2] ^1H NMR (500 MHz, $CDCl_3$) δ 7.64 – 7.56 (m, 2H), 7.45 – 7.37 (m, 2H), 7.25 – 7.20 (m, 1H), 7.11 – 7.04 (m, 2H), 7.04 – 6.96 (m, 2H).

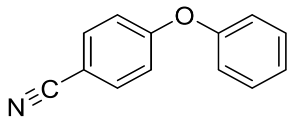

4-Tolyl phenyl ether: Spectral data are consistent with those reported in the literature.[2] The product was obtained with 63% yield. ^1H NMR (500 MHz, $CDCl_3$) δ 7.35 – 7.28 (m, 2H), 7.14 (d, *J* = 8.1 Hz, 2H), 7.07 (tt, *J* = 7.4, 1.1 Hz, 1H), 7.02 – 6.96 (m, 2H), 6.96 – 6.88 (m, 2H), 2.34 (s, 3H).

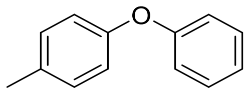

4-Phenoxybenzaldehyde: Spectral data are consistent with those reported in the literature.[3] The product was obtained with 38% yield. ^1H NMR (500 MHz, $CDCl_3$) δ 9.92 (s, 1H), 7.88 – 7.79 (m, 2H), 7.45 – 7.37 (m, 2H), 7.23 (ddt, *J* = 8.6, 7.4, 1.2 Hz, 1H), 7.12 – 7.03 (m, 4H).

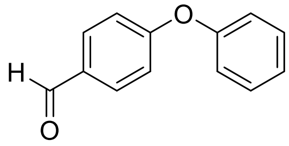

1-Methoxy-4-phenoxybenzene: Spectral data are consistent with those reported in the literature.[3] The product was obtained with 66% yield. ^1H NMR (500 MHz, $CDCl_3$) δ 7.34 – 7.28 (m, 2H), 7.08 – 7.02 (m, 1H), 7.02 – 6.97 (m, 2H), 6.97 – 6.93 (m, 2H), 6.93 – 6.86 (m, 2H), 3.82 (s, 3H).

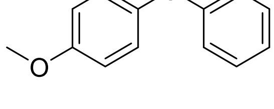

1-Phenoxy-4-(trifluoromethyl)benzene: Spectral data are consistent with those reported in the literature.[2] The product was obtained with 67% yield. ^{1}H NMR (500 MHz, $CDCl_3$) δ 7.61 – 7.53 (m, 2H), 7.43 – 7.36 (m, 2H), 7.19 (ddd, J = 8.5, 6.8, 1.1 Hz, 1H), 7.11 – 6.99 (m, 4H).

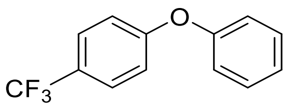

4-phenoxybenzamide: This compound was prepared following a literature procedure.[4] A 100 mL round bottom flask was charged with 1.06 g of 4-phenoxybenzonitrile (5.4 mmol), 0.112 g of K_2CO_3 (15 mol%), 3.8 mL of aqueous 30% H_2O_2 solution and 22 mL of DMSO. The mixture was stirred at 0 °C for 2 h and monitored by TLC. Upon completion, the mixture was diluted slowly with DI water and the precipitate was filtered and washed with DI water. The crude product was recrystallized from EtOH/H_2O (1:1) to afford a white solid product in 55% yield. Spectral data are consistent with those reported in the literature.[4] ^{1}H NMR (500 MHz, $CDCl_3$) δ 7.84 – 7.76 (m, 2H), 7.44 – 7.34 (m, 2H), 7.23 – 7.15 (m, 1H), 7.10 – 7.05 (m, 2H), 7.05 – 6.99 (m, 2H), 6.23 – 5.35 (m, 2H).

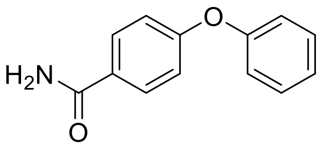

1-(Phenoxymethyl)-4-(trifluoromethyl)benzene: This compound was prepared according to a literature procedure.[5] 4-(trifluoromethyl)benzyl bromide (2 g, 8.37 mmol) was added to a mixture of phenol (0.866 g, 8.37 mmol; 1.1 equiv.) and K_2CO_3 (1.39 g, 10.06 mmol) in acetone (60 mL). The mixture was stirred at room temperature for 24 h. The reaction was filtered with Whatman paper, the solvent removed by rotary evaporation, and the resulting residue was purified by column chromatography with DCM/pentane (4:1 and then 3:7) to afford the product (1.69 g) in 76% yield. Spectral data are consistent with those reported in the literature.[6] ^{1}H NMR (500 MHz, $CDCl_3$) δ 7.70 – 7.61 (m, 2H), 7.61 – 7.53 (m, 2H), 7.36 – 7.29 (m, 2H), 7.04 – 6.92 (m, 3H), 5.14 (d, J = 1.2 Hz, 2H).

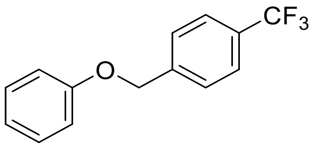

Synthetic Polymer Protocol: The following polymers were prepared using a literature procedure.[7] Br-acetophenone monomer (500 mg, 2.0 mmol) was stirred in anhydrous DMF (2.5 mL) under a N_2 atmosphere. Dry powdered K_2CO_3 (422 mg, 3.0 mmol) was added to the mixture and the reaction was stirred at 50 °C overnight. The mixture was poured into ice water (100 mL), filtered, and washed with water and methanol. The remaining solids were frozen and lyophilized to afford an insoluble dry powder. Further reduction of the synthetic polymer was achieved as

follows: The polymer (200 mg) was stirred in DMSO (10 mL). To the suspension, $NaBH_4$ (230 mg) was added and the reaction was heated to 50 °C under N_2 atmosphere overnight. The reaction mixture was poured into ice water (200 mL) and acidified to pH 3 with HCl. The slurry was filtered, washed with water, frozen, and lyophilized. The dry powder was stirred with dioxane and poured into diethyl ether to remove low molecular weight compounds. The mixture was filtered, and the solids were frozen and lyophilized to afford a dry powder. The bromine content of the polymers was analyzed by Midwest Microlab (Indianapolis, IN) and used to calculate the approximate polymer molecular weight of the insoluble polymers.

Polymer A Polymer B Polymer C

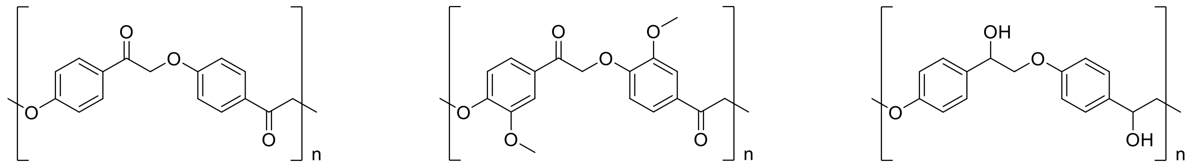

Lignin Extraction Protocol: Lignin was prepared from 18-year-old debarked (*Populus nigra* var. *charkoviensis* x *caudina* cv. NE-19) hybrid poplar (harvested in 2011 from University of Wisconsin-Madison, Arlington plots) that was chipped and hammer-milled to 5 mm grind size. Lignin was isolated according to previous procedures using copper-catalyzed alkaline hydrogen peroxide (Cu-AHP pretreatment).[9] Briefly, poplar (100 g) was incubated with aqueous NaOH (270 mM) at 10% solid loadings (1 liter) at 30 °C shaking for 1 h. The biomass was washed with deionized water (500 mL) and pretreated for an additional 23 h. at 10% solid loadings (1 liter) with additional NaOH (270 mM), copper (1 mM), bipyridine (2 mM), and hydrogen peroxide (100 mg H_2O_2 per g of biomass added over the course of 10 h). The mixture was then filtered to remove the solid biomass, and acid insoluble lignin was precipitated from the filtrate by lowering the pH to 2 with H_2SO_4. The precipitated lignin was centrifuged and washed 3 times with pH 2 water, frozen, and lyophilized yielding a light-yellow powder. This powder was then used for depolymerization studies.

III. General Procedure for Cleavage Reactions and Analysis

i. Dimers

Lignin model dimers (10 mg) and dried powdered K_2CO_3 (~100 mg) were added to an oven dried round-bottom flask (50 mL) equipped with a stir bar, condenser, septum, and nitrogen-filled balloon, and the entire apparatus was purged with N_2. Acetonitrile (20 mL) and thiol (10 equiv. to dimer) were added through a septum, and the reaction was refluxed with periodic sampling for HPLC analysis to monitor progress over 24 h.

ii. Synthetic Polymer

Synthetic polymer (10 mg), dried powdered K_2CO_3 (100 mg), and thiol (0.5 mL) were added to a glass vial (~5 mL) and closed with an aluminum cap. The mixture was stirred at 100 °C in an oil bath for 3 h. The solution was dissolved in 20 mL of water and analyzed by LCMS according to the specifications written out in the General Information section above. For sulfur analysis, the reaction was scaled up 5x and the depolymerized thiol-treated solution was centrifuged, decanted, and the remaining solid was washed 3x with alkaline water. This washed solid was frozen, lyophilized, and the dry powdered was sent to A&L Great Lakes (Fort Wayne, IN) for sulfur analysis.

iii. Lignin

Thiol-treated lignin was treated and processed as follows unless otherwise noted: Lignin (100 mg) and dried powdered K_2CO_3 (100 mg) was added to a glass vial (~10 mL) with a stir bar, capped with a septum, and purged with nitrogen. Thiol (1 mL) was added to the reaction and capped with an aluminum covered cap and stirred at 100 °C in an oil bath for 1-24 h.

HPLC: For molecular weight analysis, the resulting mixture was cooled to room temp, dissolved in 20 mL of water, centrifuged, and injected directly to the HPLC-GPC using the conditions stated in the General Information section. Lignin, unreacted with thiol, was worked up in the same manner (100 mg of lignin dissolved in 20 mL of water with 100 mg of K_2CO_3) and injected directly to the HPLC as a control comparison. Retention times were compared to a sodium polystyrene sulfonate kit from Scientific Polymer Products (M_n 1,440-85,600 g/mol). The molecular weight at the highest peak (M_p) was calculated directly from external standards while the number average molecular weight (M_n), weight average molecular weight (M_w), and polydispersity (PDI) were calculated from the following equations (1-3):

$$M_n = \frac{\sum N_i M_i}{\sum N_i} \quad (1)$$

$$M_w = \frac{\sum N_i M_i^2}{\sum N_i M_i} \quad (2)$$

$$PDI = \frac{M_w}{M_n} \quad (3)$$

where N_i represents the total number of different molecular weights chains and M_i is the molecular weight of each polymer chain.

For elemental analysis, mass balance, thioacidolysis, and NMR characterization, the aqueous solubilized thiol-treated lignin was precipitated with H_2SO_4 (HCl for sulfur analysis) to pH 2, centrifuged and washed with acidic water, frozen, and lyophilized. The resulting dry depolymerized lignin was then characterized by the following:

NMR:

For 2-D NMR analysis on lignin before and after depolymerization, gHSQC-AD NMR was used according to literature procedure.[8] Briefly, dry lignin (40 mg) was dissolved in 500 μL of DMSO-d^6. The solution was added to an NMR tube and run with either a Varian 600 MHz or a Bruker 900 MHz NMR.

The Bruker BioSpin Avance GmbH 900 MHz superconducting NMR-Spectrometer equipped with a TCI triple resonance inverse detection Cryoprobe, 5 mm CPTCI 1H-13C/ 15N/ D Z-GRD Z44910/ 0007 used the following parameters: DMSO-d^6 solvent peak was used as an internal reference (δ_C 39.5, δ_H 2.49 ppm); The ^{13}C-^{1}H correlation experiment was adiabatic gHSQC-AD using the pulse sequence hsqcedetgpsisp2.2 with spectra acquired from 13 to -3 ppm in F2 (^{1}H) using a pulse width of 9.25 sec, a relaxation delay of 1.5 sec, acquisition time 0.0713 sec, F2 (^{1}H) spectral width of 14367.8, spectral and acquired size of 1024; 200 to 0 ppm in F1 (^{13}C) using a spectral width of 45196.2, acquired size 400, and a spectral size of 1024; 32 scans, with a total acquisition time of 16 h.

The Varian Inova 600 MHz superconducting NMR-Spectrometer operating at 599.892 MHz, running CentOS 5.6 with VnmrJ 3.2A and equipped with an HCN probe used the following parameters: DMSO-d^6 solvent peak was used as an internal reference (39.5, 2.49 ppm); The ^{13}C-^{1}H correlation experiment was adiabatic gHSQC-AD using the standard gHSQCAD pulse sequence with spectra acquired from 14 to -2 ppm in F2 (^{1}H) using a pulse width of 8.5 sec, a

relaxation delay of 1.0 sec, acquisition time 0.15 sec, F2 (^{1}H) spectral width of 9595.8, spectral size of 2048, and an acquired size of 1439; 190 to -10 ppm in F1 (^{13}C) using a spectral width of 20155.9, acquired size 512, and a spectral size 2048; 72 scans, with a total acquisition time of 24 h.

For -OH content of lignin before and after depolymerization, ^{31}P NMR was used according to literature procedure.[9] Briefly, lignin (40 mg) was dissolved in 500 µL of anhydrous pyridine/deuterated chloroform solution (1.6:1, v/v) and 100 µL of cyclohexanol. To this solution 50 µL of chromium acetylacetonate solution (5.6 mg/mL in anhydrous pyridine/deuterated chloroform solution (1.6:1, v/v)) and 100 µL of 2-chloro-4,4,5,5-tetramethyl 1,3,2-dioxaphospholane were added and vortexed until fully dissolved. The solution was added to an NMR tube and run with a 500 MHz Agilent DDR2 NMR equipped with a phosphorous probe using a 45° pulse angle, 5 sec pulse delay, and 512 scans. Peaks were integrated using the internal standard, cyclohexanol.

Mass balance: The dry solids remaining were weighed and compared to the weight of lignin un-reacted with thiol, that was worked up in the same manner (dissolved in base and water and acidified, centrifuged, washed with acidic water, frozen, and lyophilized).

Elemental analysis: Samples were analyzed by A & L Great Lakes (Fort Wayne, IN) for sulfur content.

Thioacidolysis: Samples were analyzed by the GLBRC-Core Facility (Michigan State University- East Lansing, MI) for S:G ratio.[10]

iv. Lignin Oxidation

Cu-AHP lignin (1.5 g) and Bobbitt's salt (4-acetamido-2,2,6,6-tetramethyl-1-oxopiperidinium tetrafluoroborate) (3.5 g) were suspended in 150 mL of acetonitrile/water mixture (4:1, v/v), and 15 mL of 0.5 M HCl were added under stirring. The suspension was refluxed at 80 °C for 2 h with magnetic stirring at 500 rpm. After cooling to room temperature, the pH of the resulting solution was adjusted to 2.0 by 0.1 M H_2SO_4 and the suspension was stored in refrigerator overnight. The precipitated lignin was isolated by centrifugation at 3000 rpm, 4 °C for 30 min. Collected lignin was washed with 100 mL deionized water, centrifuged again and freeze-dried.

IV. Cleavage of 4-O-5 Models

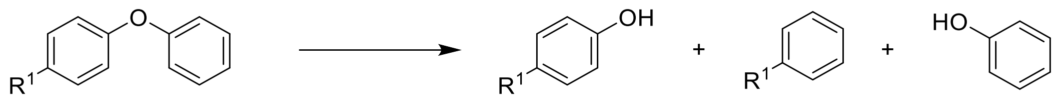

1	R^1 = H 0%	0%	-	0%
2	R^1 = OMe 1%	0%	0%	0%
3	R^1 = Me 1%	0%	-	0%
4	R^1 = OH 4%	0%	-	3%
5	R^1 = CF_3 0%	0%	0%	0%
6	R^1 = $COCH_3$ 9%	7%	0%	7%
7	R^1 = CN 21%	0%	-	-
8	R^1 =CHO 85%	2%	0%	78%
9	R^1 =$CONH_2$ 5%	-	-	0%

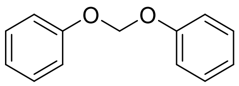

No RXN

0% diphenoxymethane

Table S1. Cleavage yields of 4-O-5 dimers, a non-β-O-4 ether linkage of lignin. Reactions were carried out using 10 equiv. of 2-mercaptoethanol (BME) in refluxing MeCN with stirring K_2CO_3 for 24 h. Conversion is shown for substrate and yields are given for products. Yields were assessed by phenolic product. Benzene (1) and toluene (3) were not quantified due to insolubility in direct injection HPLC. (7) was cleaved but quantification of phenol and benzonitrile was unsuccessful due to chromatography separation. (9) No phenol product was produced with no external standards for 4-hydroxybenzamide or benzamide. Only the aldehyde (8) and ketone (6) substituted 4-O-5 dimers were cleaved successfully suggesting that a carbonyl is needed to activate a nucleophilic aromatic substitution using thiols. No cleavage was observed for diphenoxymethane.

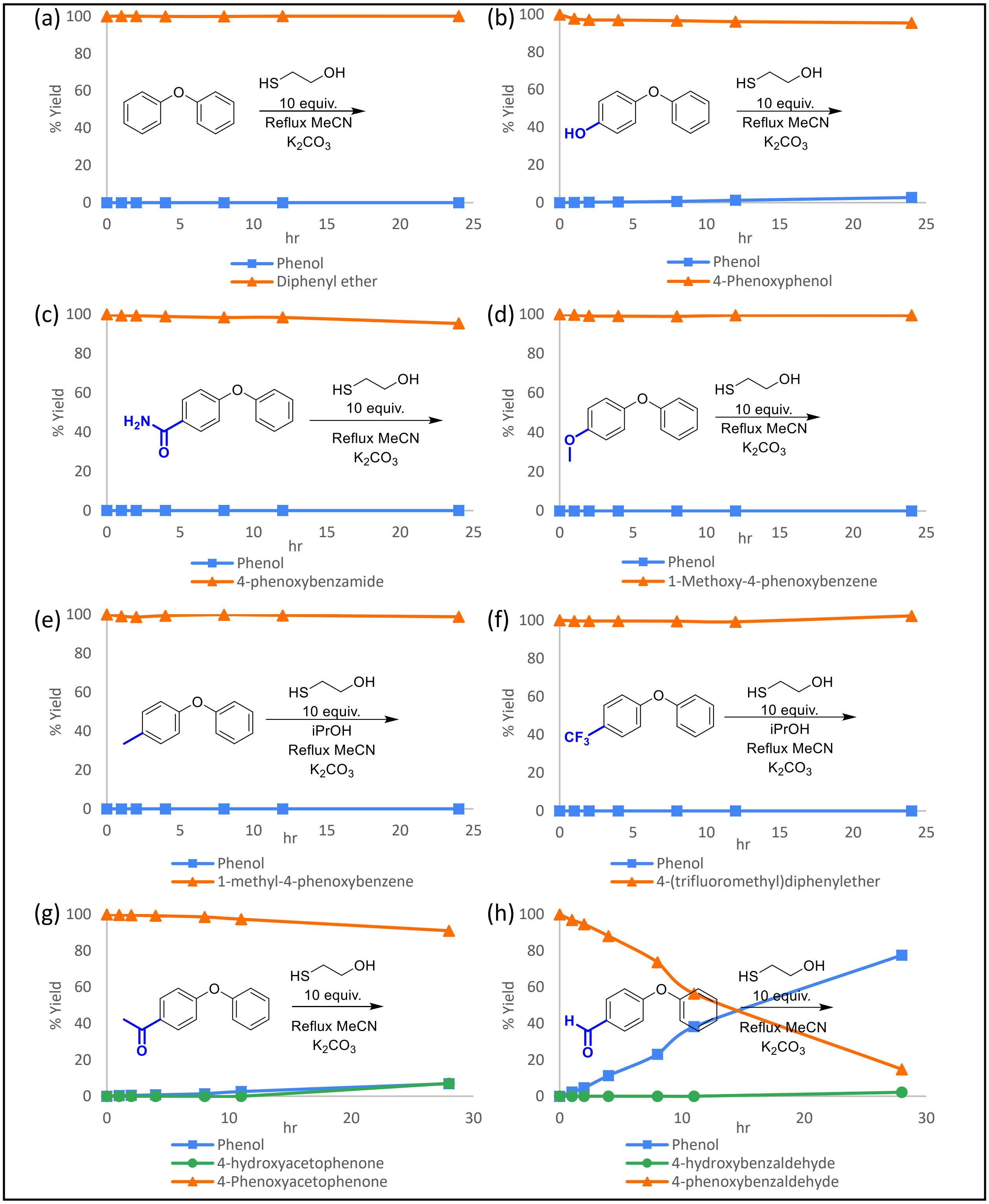

Figure S1. 4-O-5 dimer cleavage using 10 equiv. of 2-mercaptoethanol (BME) and K_2CO_3 while stirring with refluxing MeCN for 24 h. Very little cleavage is seen except in the case of (h) where the *p*-directing aldehyde promotes nucleophilic aromatic substitution, releasing phenol. The corresponding implied thioether product was not quantified.

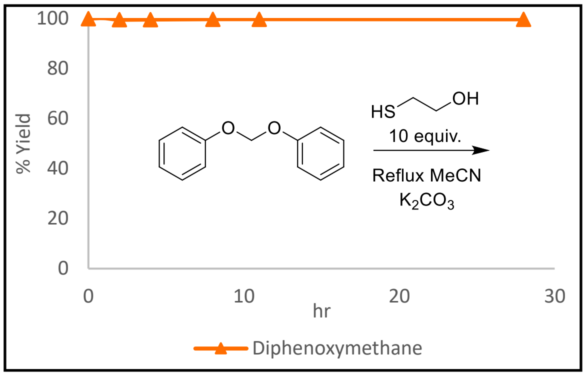

Figure S2. Thiol-mediated cleavage of other aryl ethers. Diphenoxymethane was subjected to 10 equiv. of 2-mercaptoethanol (BME) and K_2CO_3 under refluxing MeCN for 24 h. but did not undergo cleavage. This control reaction supports the need of a carbonyl to promote ether cleavage using small thiols.

V. Cleavage of α-O-4/β-5 Models

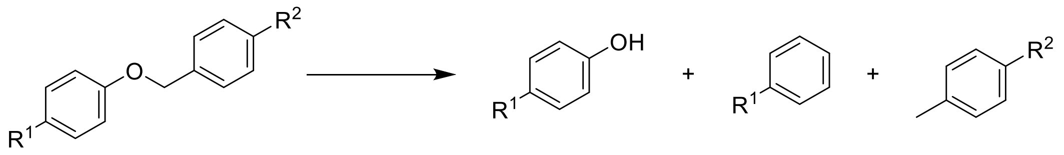

1	R^1 = H; R^2 = H 0%	0%	0%	0%
2	R^1 = NH_2; R^2 = H 2%	-	0%	-
3	R^1 = OH; R^2 = H 6%	0%	0%	0%
4	R^1 = CN; R^2 = H 12%	2%	0%	0%
5	R^1 = ring; R^2 = H 0%	0%	0%	0%
6	R^1 = CF_3; R^2 = H 0%	0%	0%	0%
7	R^1 = H; R^2 = CF3 1%	0%	0%	0%
8	R^1 = H; R^2 = CHO 49%	25%	0%	0%
9	R^1 = CHO; R^2 = H 13%	2%	0%	0%

Table S2. Cleavage yields of α-O-4/β-5 dimers, a non-β-O-4 ether linkage of lignin. Reactions were carried out using 10 equiv. of 2-mercaptoethanol (BME) in refluxing MeCN with stirring K_2CO_3 for 24 h. Conversion is shown for substrate and yields are given for products. Yields were assessed by phenolic product. Benzene and toluene were not quantified due to insolubility in direct injection HPLC. (2) No phenol product was produced with no external standard for 4-aminophenol. Similar to the 4-O-5 dimer cleavage, α-O-4/β-5 dimers activated with an aldehyde enable cleavage through a possible S_N2 mechanism.

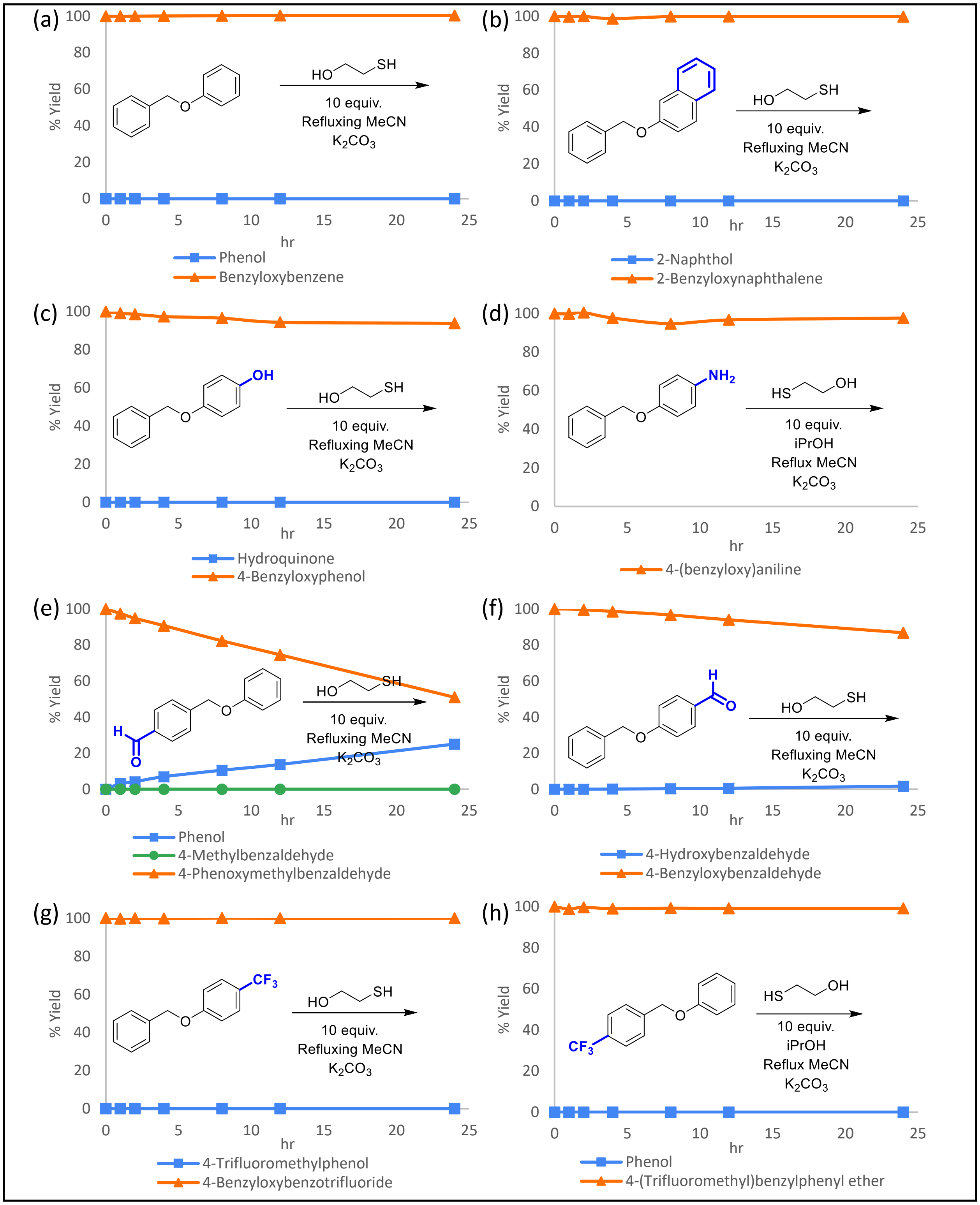

Figure S3. α-O-4/β-5 dimer cleavage using 10 equiv. of 2-mercaptoethanol (BME) and stirring K_2CO_3 with refluxing MeCN for 24 h. Very little cleavage is seen except (e) where the *p*-directing aldehyde promotes nucleophilic attack on the C_α-position, releasing a phenol product. (f) resulted in <5% cleavage suggesting that the electronics of the α-position are more important for cleavage.

VI. Dimer Control Experiments

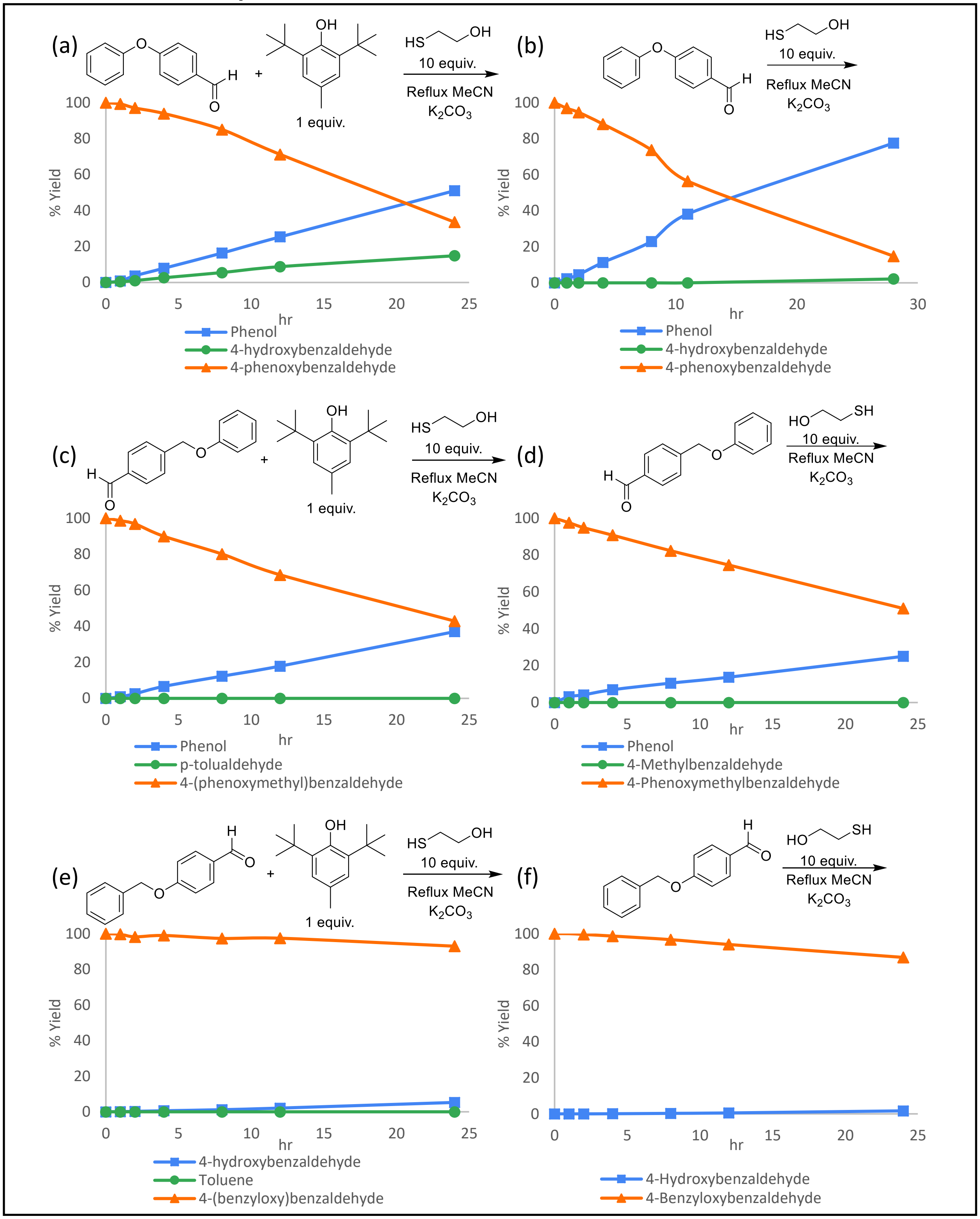

Figure S4. Control reactions to determine a non-radical mechanism for the cleavage of 4-O-5 and α-O-4/β-5 dimers with *p*-directing aldehydes. (a), (c), and (e) on the left side were reacted

with 10 equiv. of 2-mercaptoethanol (BME) in stirring K_2CO_3 with refluxing MeCN and the addition of 1 equiv. of BHT, a known radical scavenger. (b), (d), and (f) on the right side were subjected to the same conditions but without BHT. Very little difference in yield is seen between the 3 separate substrates and their corresponding BHT control reaction. This suggests that the cleavage mechanism does not involve a free radical.

VII. Proposed Dimer Cleavage Mechanism

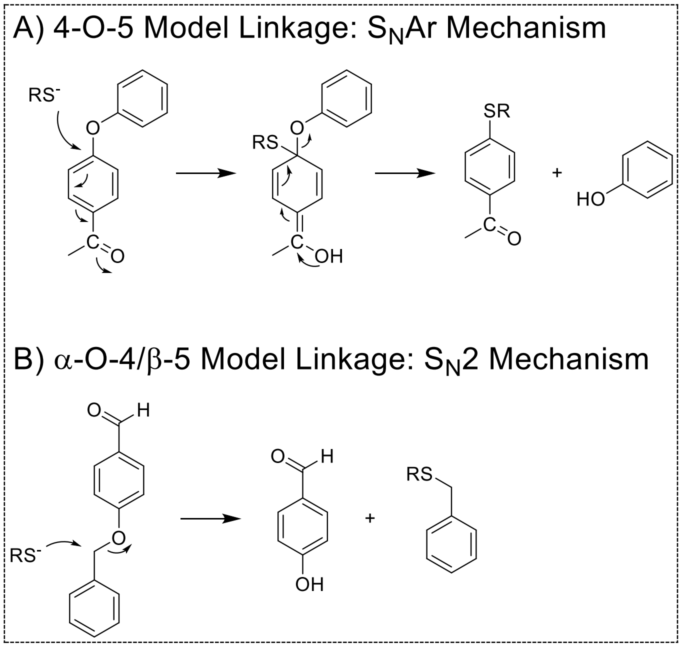

Figure S5. A) Proposed model 4-O-5 ether cleavage via thiol mediated nucleophilic aromatic substitution. B) Proposed model α-O-4/β-5 ether cleavage via an S_N2 mechanism activated by the *p*-aldehyde.

VIII. Synthetic Polymer Cleavage

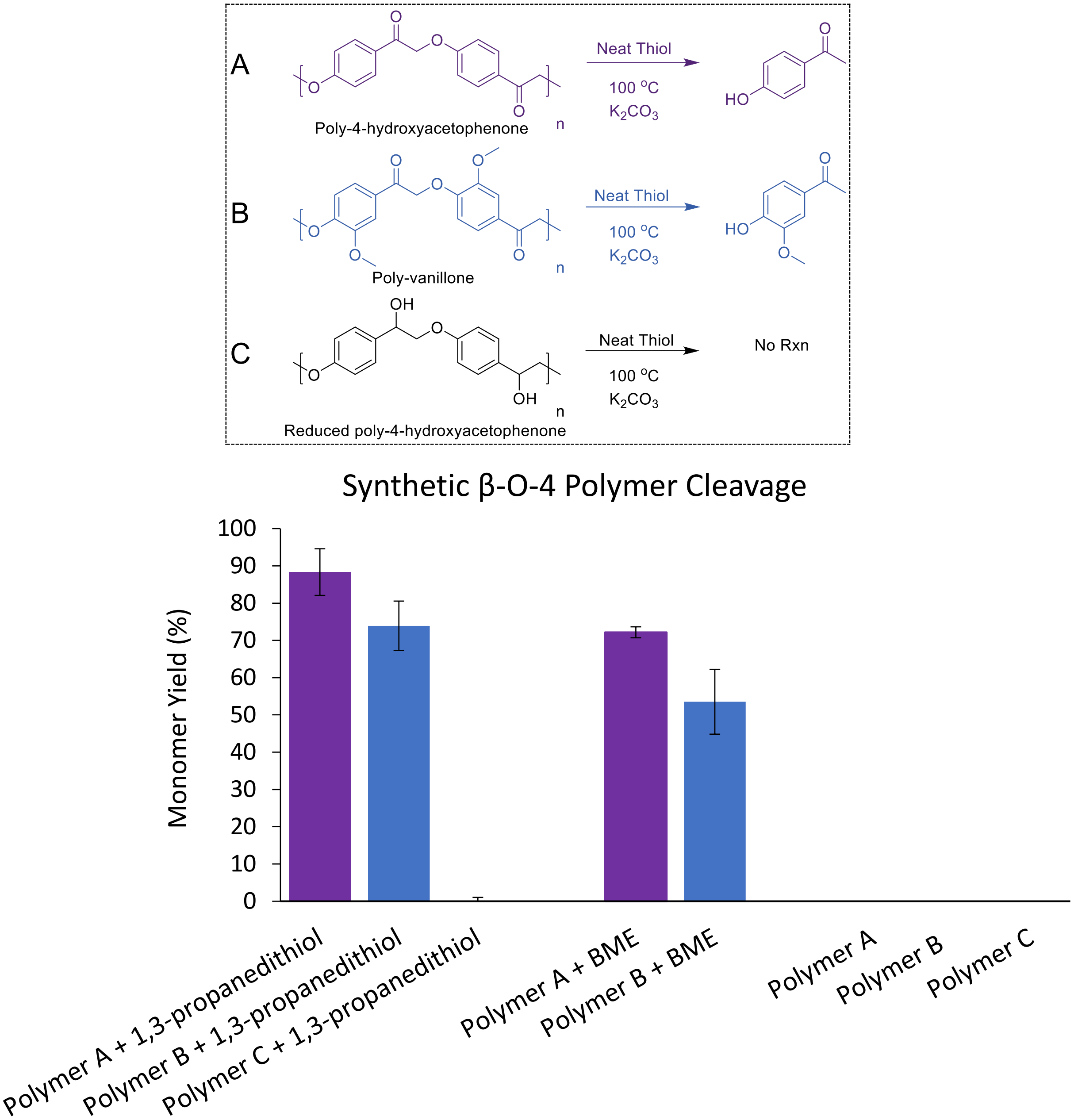

Figure S6. Thiol-mediated cleavage of synthetic β-O-4 lignin. Monomer cleavage yields are shown (bottom) for synthetic β-O-4 polymers shown (top) using neat 1,3-propanedithiol or neat 2-mercaptoethanol (BME) in stirring K_2CO_3 at 100 °C for 3 h. These monomer yields are compared to yields without addition of thiol. Polymer A (purple) and B (blue) have high cleavage

yields, >70%, when treated with 1,3-propanedithiol. However, no monomer products are seen from the reaction without the addition of thiol. Furthermore, Polymer C, the reduced version of Polymer A, did not yield any monomer products whether subjected to thiol treatment or not. These results support the ability of thiols such as BME and 1,3-propanedithiol to diffuse through bulky polymeric systems and cleave β-O-4 bonds. These results also give additional evidence that the α-position must be oxidized, as seen in previous dimer studies[11] and in the enzymatic pathway,[12] in order for thiols to nucleophilically attack the β-aryl ether site. Reactions were performed in triplicate.

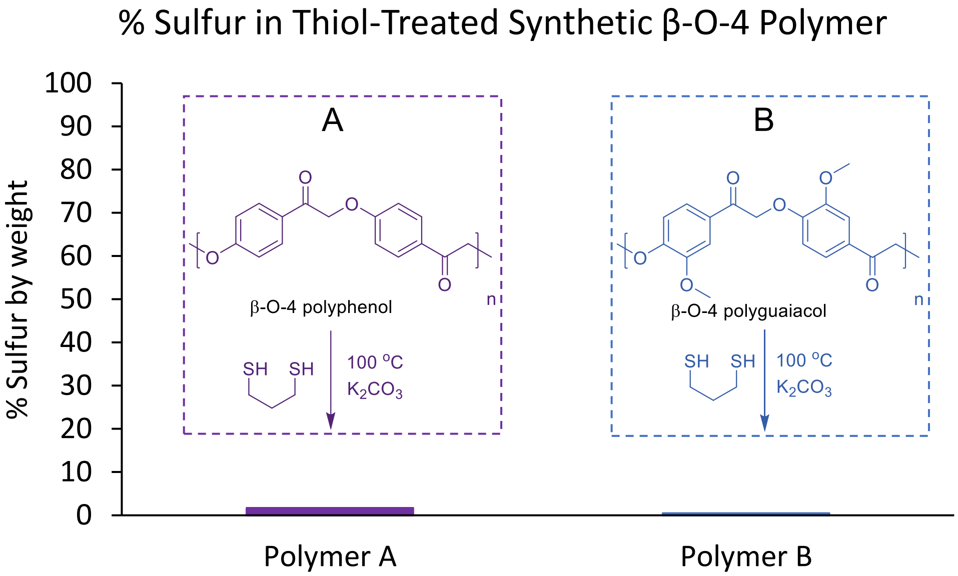

Figure S7. Analysis of sulfur incorporated into depolymerized synthetic β-O-4 lignin. Elemental analysis of the remaining solids of synthetic β-O-4 polymer after depolymerization with 1,3-propanedithiol and K_2CO_3 at 100 °C for 24 h. Roughly 22% of polymer A (purple) remained uncleaved after thiol mediated depolymerization and from that 22%, there was 1.693% sulfur content or 1.86 mg of sulfur remaining. Roughly 13.6% of polymer B (blue) remained uncleaved after thiol mediated depolymerization and from that 13.6%, there was 0.514% sulfur content or 0.350 mg of sulfur remaining. The low percent of sulfur suggests that the remaining unreacted polymer successfully completed the first step of the S_N2 reaction, forming a thioether, but stopped before forming the disulfide and releasing the second monomer.

IX. Lignin Polymer Model

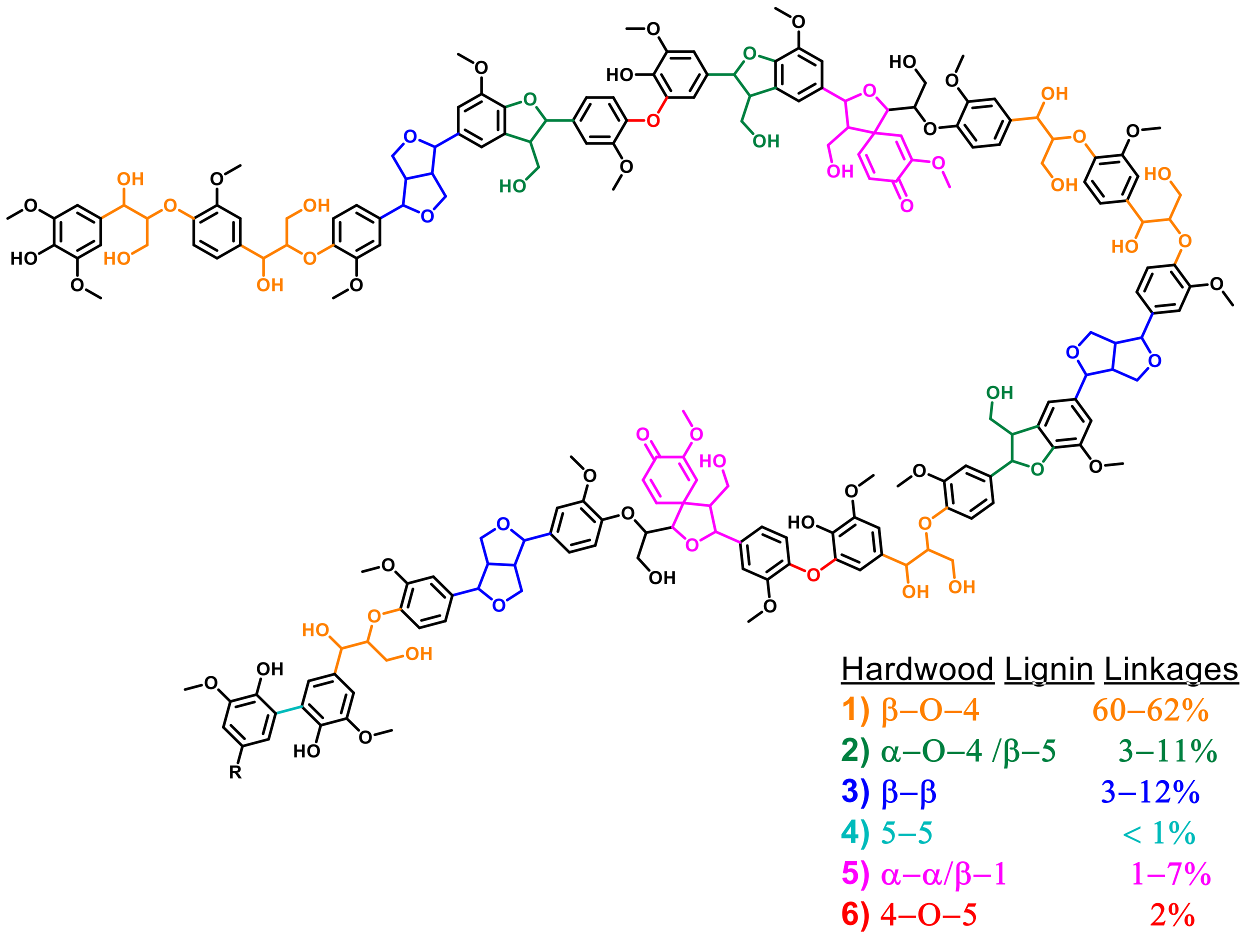

Figure S8. Representation of general hardwood lignin and its common linkages highlighted by color. The most common linkage is the β-O-4 bond (highlighted in orange) with other ether bonds highlighted in red (4-O-5) and green (α-O-4/β-5). The percent of the various linkages in lignin is based on hardwoods.[13] In poplar lignin, a small amount of *p*-hydroxybenzoic acid is present on the gamma hydroxyl groups of S-units, but they are largely removed during the Cu-AHP (copper catalyzed alkaline hydrogen peroxide pretreatment) lignin isolation process.

X. Lignin Cleavage

(a)

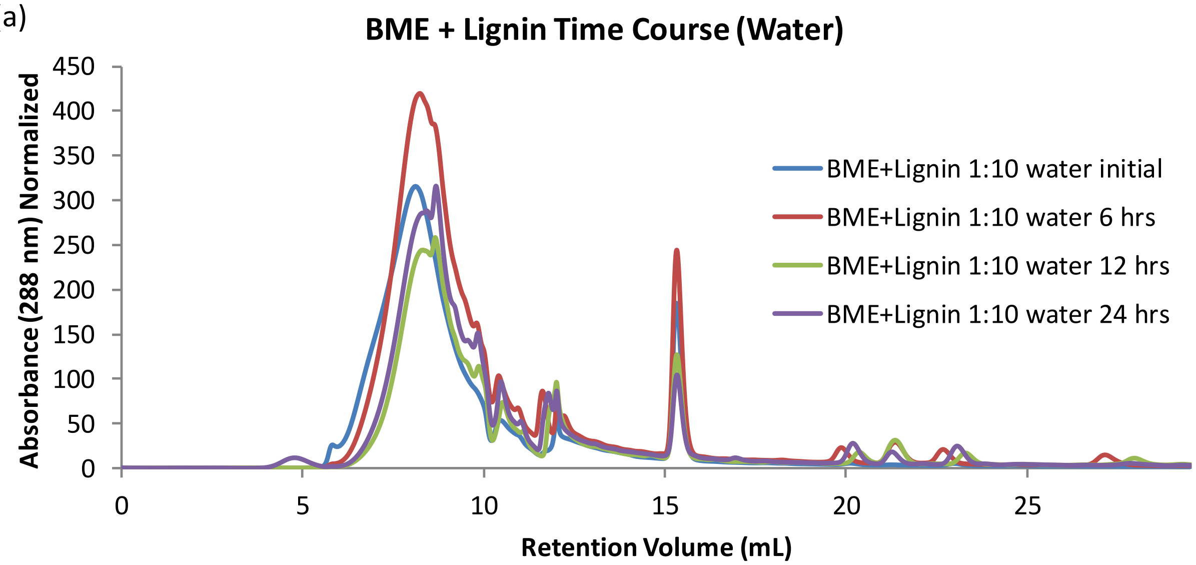

(b)

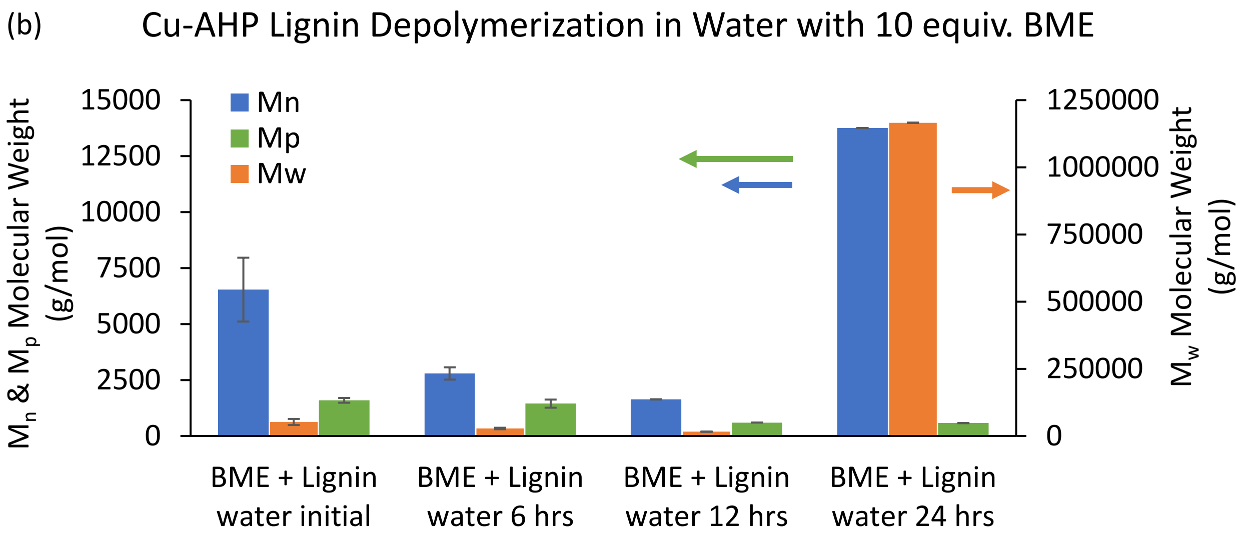

Figure S9. Thiol-mediated Cu-AHP lignin depolymerization in water. Molecular weight comparisons for Cu-AHP lignin treated with 10 equiv. of 2-mercaptoethanol (BME) and stirring K_2CO_3 in refluxing water for 0, 6, 12, and 24 h. (a) Gel permeation chromatogram of molecular weight distributions of Cu-AHP lignin at different reaction times (0 h, blue; 6 h, red; 12 h, green; and 24 h, purple). (b) Calculated molecular weight comparisons for M_n in blue (number average molecular weight), M_w in orange (weight average molecular weight), and M_p in green (high peak molecular weight) for 0, 6, 12, and 24 h. M_n and M_p axis is shown on the left and M_w axis is shown on the right. Visually, from the chromatogram and the calculated values, lignin depolymerization does not work well in aqueous conditions, similar to what was found in our dimer studies.[11] Reactions were performed in duplicate.

(a)

Cu-AHP Lignin Depolymerization

Conditions	Mass Change (%)	Conditions	Mass Change (%)
DTT in DMF	-65.2 ± 5.09	BME in DMF	-82.5 ± 10.61
DTT in DMSO	+62.15 ± 15.34	BME in DMSO	+116.25 ± 68.66
DTT in NMP	-75.5 ± 1.13	BME in NMP	-86.35 ± 1.77
DTT in H_2O	-47.75 ± 8.56	BME in H_2O	+111.6 ± 77.22
DTT in H_2O and NaOH	+46.1 ± 58.55	BME in H_2O and NaOH	+59.3 ± 53.60

(b)

Cu-AHP Lignin Depolymerization

Conditions	M_p	Conditions	M_p
DTT in DMF	6,929	BME in DMF	6,182
DTT in DMSO	1,772	BME in DMSO	1,767
DTT in NMP	7,333	BME in NMP	6,726
DTT in H_2O	6,041	BME in H_2O	5,364
DTT in H_2O (NaOH)	927	BME in H_2O (NaOH)	933
Cu-AHP Lignin	5,311		

(c)

Cu-AHP Lignin Depolymerization

Conditions	Mass Change (%)
Neat DTT	-54.2
Neat thiophenol	+52.2
Neat BME	-64.4
Neat 1,3-propanedithiol	+62.8

Figure S10. Comparison of percent mass change variability during Cu-AHP lignin depolymerization with various solvents. (+) indicates mass gained due to thiol incorporation and/or crosslinking and (-) indicates mass loss due to possible solubilization of cleaved fragments. (a) Mass change (%) of Cu-AHP lignin reaction with DTT (dithiothreitol) or BME (2-mercaptoethanol) with stirring Na_2CO_3 at 100 °C in DMF, DMSO, NMP, H_2O, or H_2O (with NaOH instead of Na_2CO_3). The reactions were quenched with acid to pH 2, centrifuged, frozen, lyophilized, and the dry uncleaved/acid insoluble polymer was weighed. Standard deviation reported for 3 replicates. (b) M_p (molecular weight at the highest point of the lignin polymer) from GPC analysis of (a) compared with Cu-AHP lignin M_p. (c) Mass change (%) of Cu-AHP lignin reacted with neat thiol (DTT, BME, Thiophenol, or 1,3-propanedithiol) and stirring K_2CO_3 at 100 °C for 24 h. From the above tables it is clear that the lignin reacted under polar aprotic solvents (and water) is not an accurate representation of the lignin depolymerization due to the variability in the mass changes and the molecular weight changes compared to unreacted Cu-AHP lignin. Furthermore, mass loss change is evident in most cases but structural changes in lignin in solvating conditions resulted in solubility/precipitation problems after the reaction. Thiophenol and 1,3-propanedithiol increased the mass of the uncleaved polymer. Therefore, neat reaction conditions using BME or DTT appear to be ideal for lignin depolymerization.

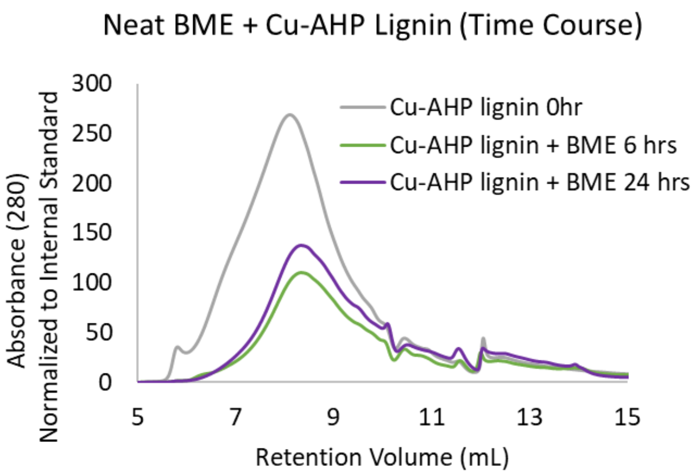

	Cu-AHP Lignin 0 h.	Cu-AHP Lignin + BME 6 h.	Cu-AHP Lignin + BME 24 h.
**M_n**	6778	2294	1954
**M_w**	55740	41726	34357
**PDI**	8.22	18.19	17.58
**M_p**	1624	1060	1051

Figure S11. Time course study to determine reaction length needed for sufficient lignin depolymerization with neat thiol. Molecular weight comparison of Cu-AHP lignin either unreacted or reacted with neat 2-mercaptoethanol (BME) for 6 or 24 h. (Top) GPC chromatogram of molecular weight distribution between Cu-AHP lignin (grey), Cu-AHP lignin treated with BME for 6 h. (green), and Cu-AHP lignin treated with BME for 24 h. (purple). (Bottom) Molecular weight comparisons for M_n (number average molecular weight), M_w (weight average molecular weight), M_p (high peak molecular weight), and PDI (polydispersity index) for the 3 time points. Cleavage of lignin after 6 h. is similar to cleavage after 24 h., suggesting that the lignin does not need to be treated with BME for as long as 24 h. to produce ether cleavage fragments.

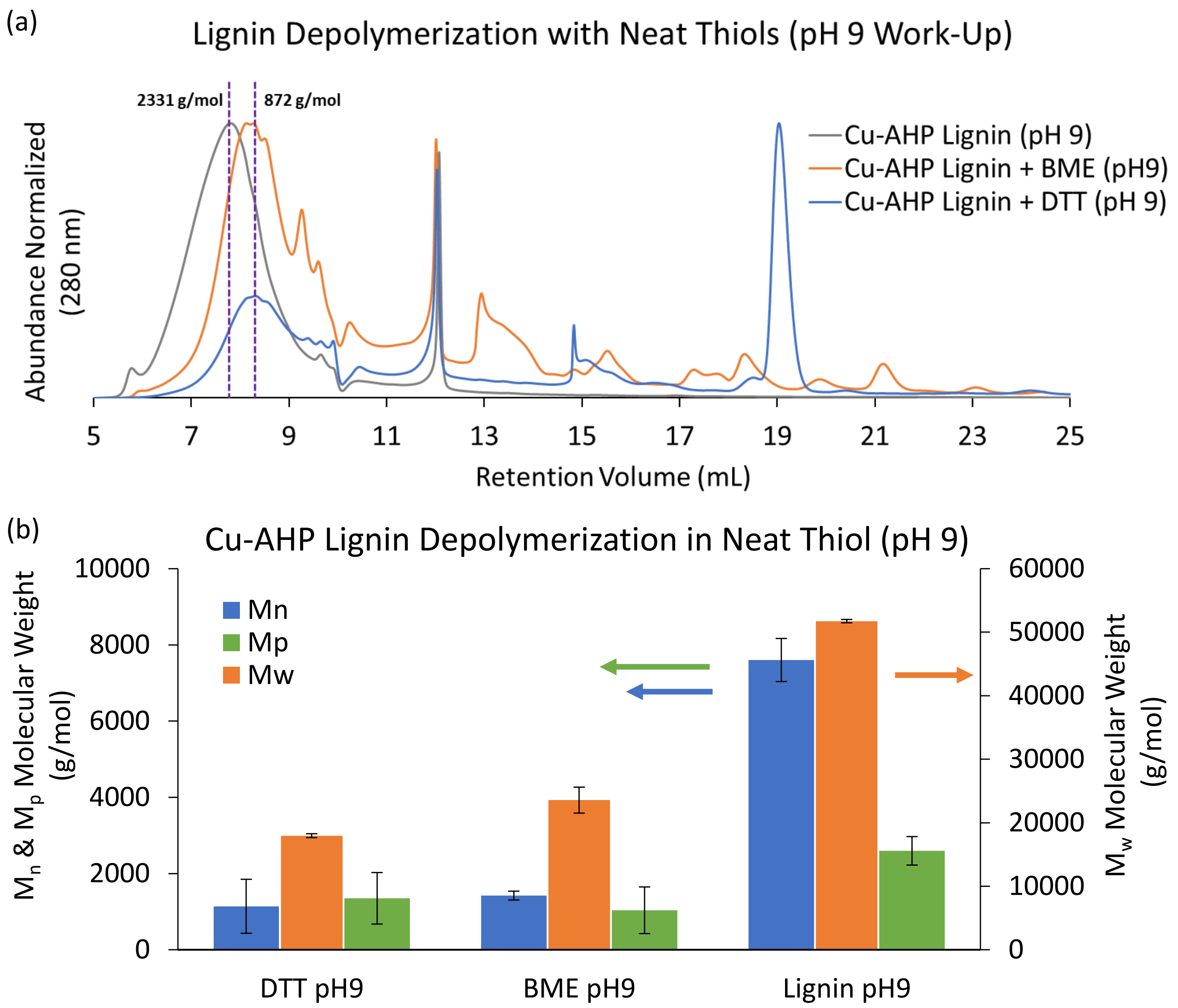

Figure S12. Polymer cleavage characterization using pH 9 workup. Molecular weight comparisons for Cu-AHP lignin treated with neat 2-mercaptoethanol (BME) or neat dithiothreitol (DTT) with stirring K_2CO_3 at 100 °C for 24 h. compared to a Cu-AHP lignin control, worked up in the same manner but without the addition of thiol. Reactions were quenched with water (20 mL) with a final pH of 9 (due to the dissolved K_2CO_3). (a) GPC chromatogram of molecular weight distributions of Cu-AHP lignin (grey), BME treated Cu-AHP lignin (orange), and DTT treated Cu-AHP lignin (blue). (b) Calculated molecular weight comparisons for M_n in blue (number average molecular weight), M_w in orange (weight average molecular weight), and M_p in green (high peak molecular weight) for Cu-AHP lignin, BME treated Cu-AHP lignin, and DTT treated Cu-AHP lignin. M_n and M_p axis is shown on the left and M_w is shown on the right. Visually, from the chromatogram and the calculated values, both BME and DTT decrease the polymer molecular weight significantly. Reactions were run in duplicate.

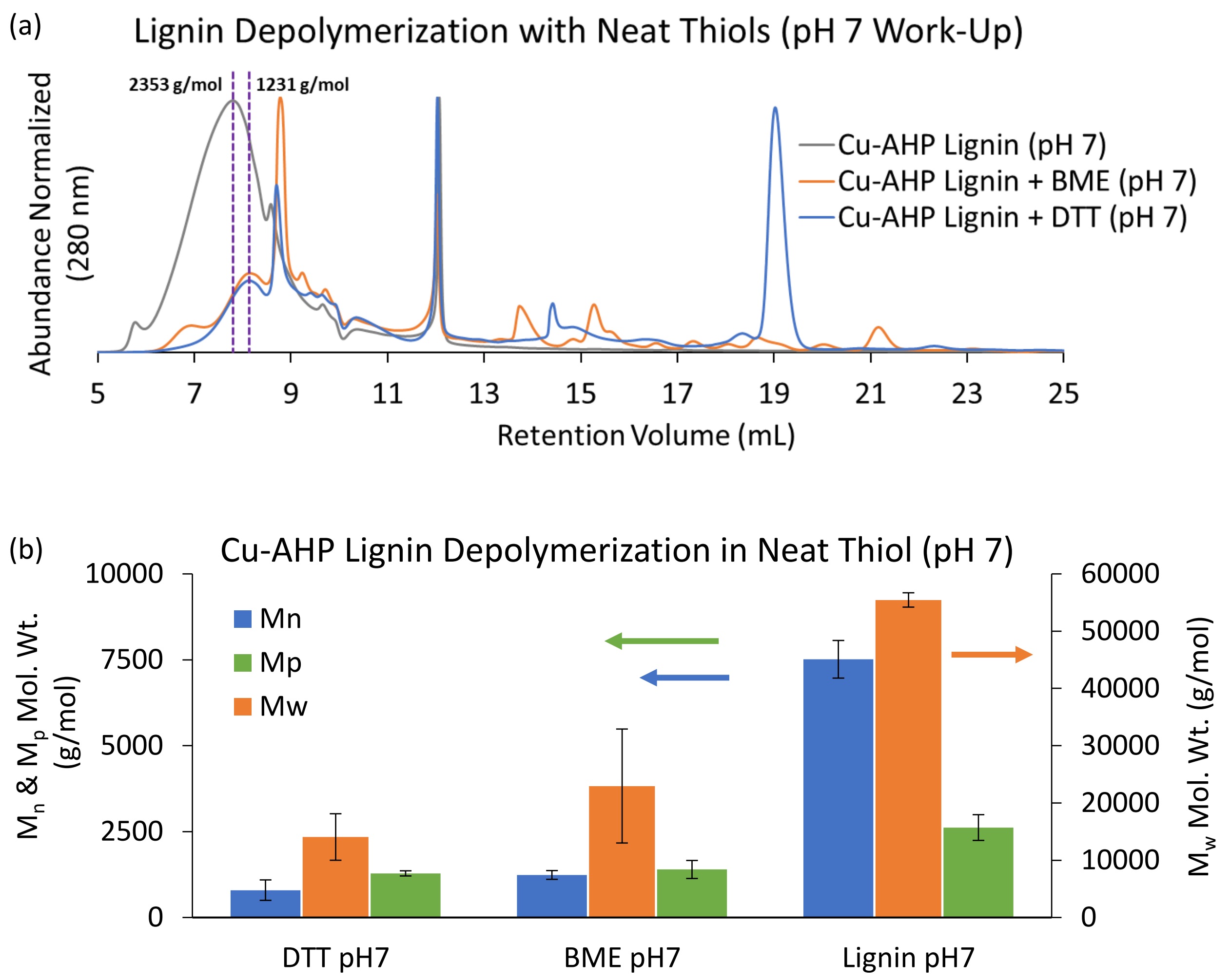

Figure S13. Polymer cleavage characterization using pH 7 workup. Molecular weight comparisons for Cu-AHP lignin treated with neat 2-mercaptoethanol (BME) or neat dithiothreitol (DTT) with stirring K_2CO_3 at 100 °C for 24 h. compared to Cu-AHP lignin worked up the same way (minus the thiol and heat) as a control. Reactions were quenched with water (20 mL) and acidified with 72% H_2SO_4 to pH 7. (a) Gel permeation chromatogram of molecular weight distributions of Cu-AHP lignin (grey), BME treated Cu-AHP lignin (orange), and DTT treated Cu-AHP lignin (blue). (b) Calculated molecular weight comparisons for M_n in blue (number average molecular weight), M_w in orange (weight average molecular weight), and M_p in green (high peak molecular weight) for Cu-AHP lignin, BME treated Cu-AHP lignin, and DTT treated Cu-AHP lignin. M_n and M_p axis is shown on the left and M_w is shown on the right. Visually, from the chromatogram and the calculated values, both BME and DTT decrease the polymer molecular weight significantly but have larger variations than the reactions analyzed directly (pH 9). Reactions were run in duplicate.

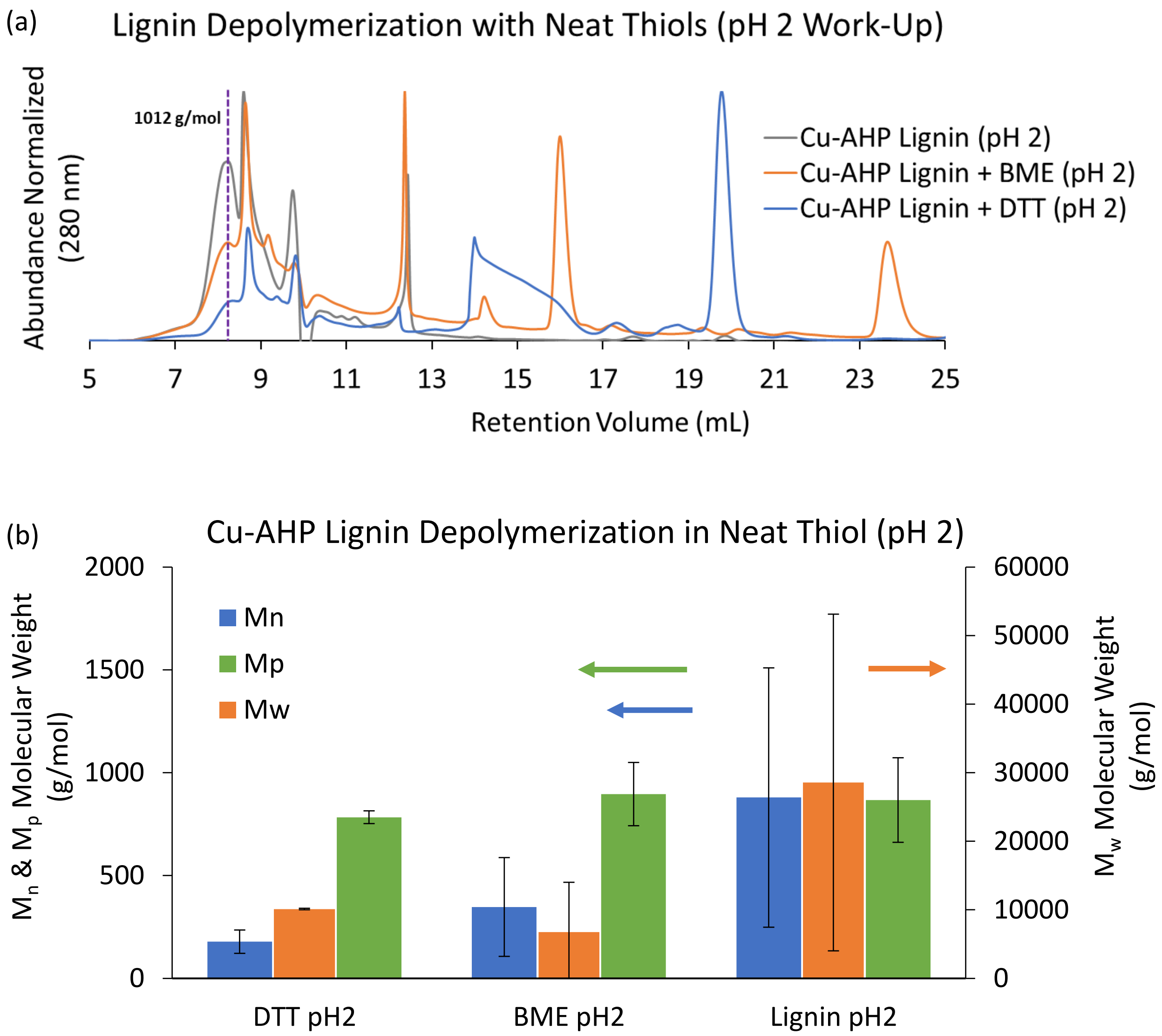

Figure S14. Polymer cleavage characterization using pH 2 workup. Molecular weight comparisons for Cu-AHP lignin treated with neat 2-mercaptoethanol (BME) or neat dithiothreitol (DTT) with stirring K_2CO_3 at 100 °C for 24 h. compared to Cu-AHP lignin worked up the same way (minus the thiol and heat) as a control. Reactions were quenched with water (20 mL) and acidified with 72% H_2SO_4 to pH 2. (a) Gel permeation chromatogram of molecular weight distributions of Cu-AHP lignin (grey), BME treated Cu-AHP lignin (orange), and DTT treated Cu-AHP lignin (blue). (b) Calculated molecular weight comparisons for M_n in blue (number average molecular weight), M_w in orange (weight average molecular weight), and M_p in green (high peak molecular weight) for Cu-AHP lignin, BME treated Cu-AHP lignin, and DTT treated Cu-AHP lignin. M_n and M_p axis is shown on the left and M_w is shown on the right. Visually, from the chromatogram and the calculated values, both BME and DTT polymer molecular weight values vary significantly with

little difference between the thiol depolymerized lignin and the lignin control. From these quenching reactions, addition of acid appears to cause large variations in molecular weight with possible precipitation. Moving forward, direct analysis of the reaction (pH 9) appears to be the most informative samples owing to the fact that the entire polymer/polymer products are dissolved and are observable and quantifiable using GPC with very little variability in replicates. Reactions were run in duplicate.

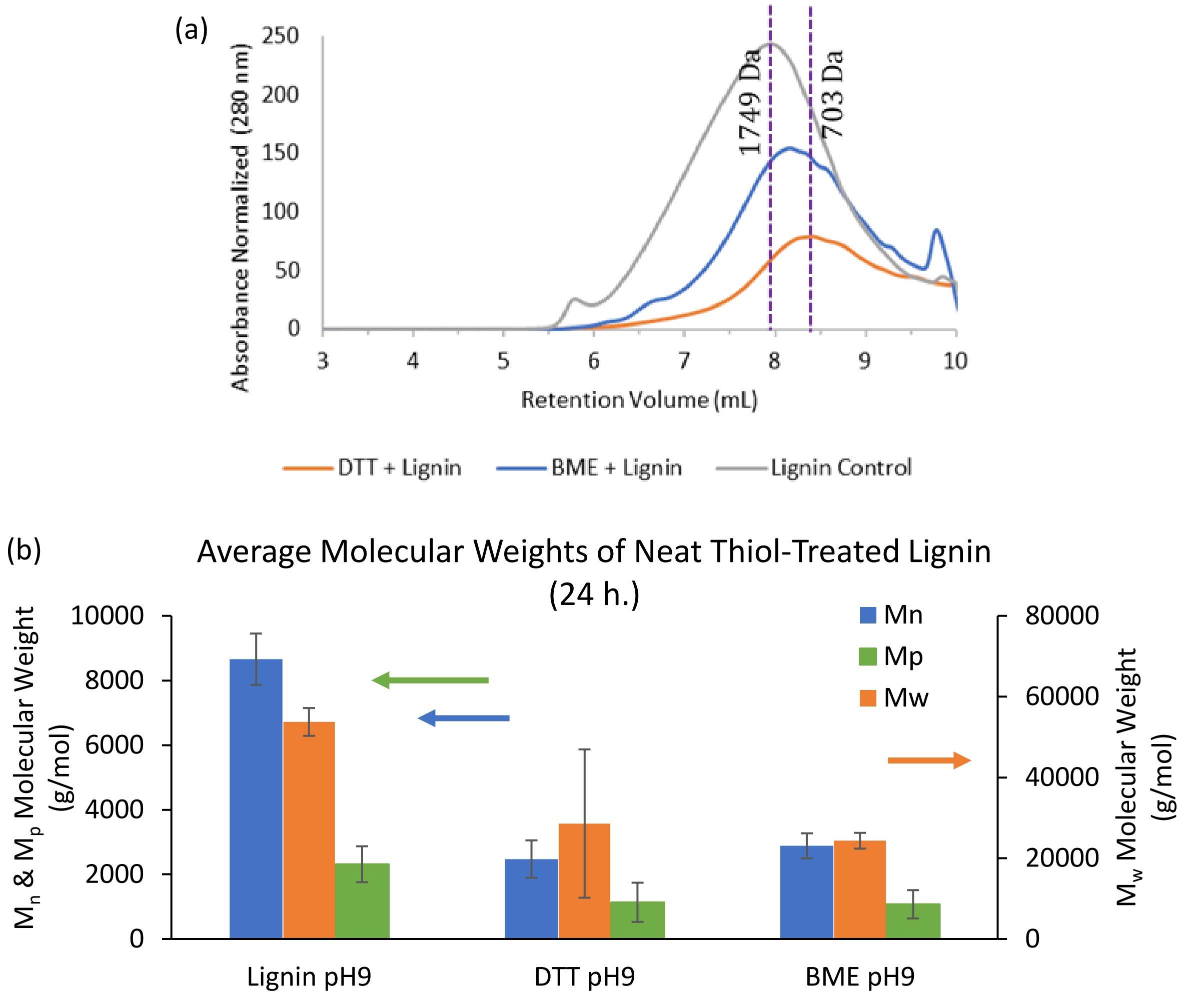

Figure S15. Depolymerization of Cu-AHP lignin with neat thiol for 24 h. worked up with water to pH 9. (a) Molecular weight distributions of untreated Cu-AHP lignin (grey), Cu-AHP lignin treated with neat dithiothreitol (DTT) (orange), and Cu-AHP lignin treated with neat 2-mercaptoethanol (BME) (blue). (b) Calculated molecular weights of M_n in blue (number average molecular weight), M_w in orange (weight average molecular weight), and M_p in green (high peak molecular weight) from the top chromatogram. M_n and M_p axis is shown on the left and M_w is shown on the right. BME-treated lignin has similar molecular weight decreases in M_n, M_w, and M_p compared to DTT-treated lignin. Reactions were run in triplicate.

(a)

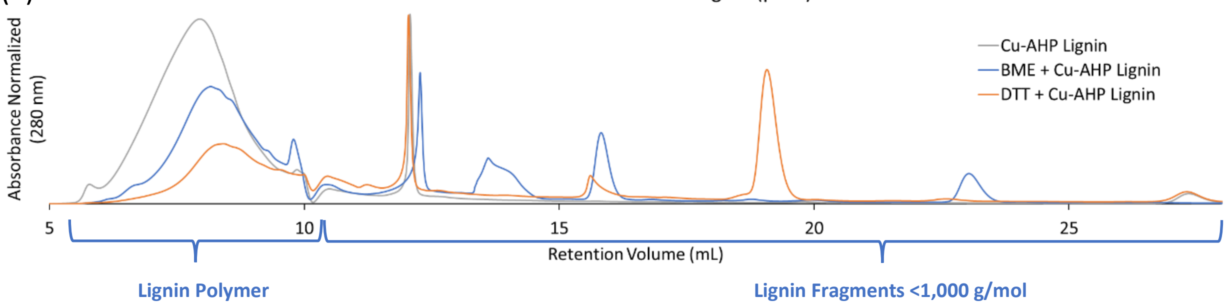

(b)

	Cu-AHP Lignin	Cu-AHP Lignin + DTT	Cu-AHP Lignin + BME
M_n	8009	2583	3208
M_w	57702	49786	25832
M_p	1749	703	1150
PDI	7.20	19.27	8.05

(c)

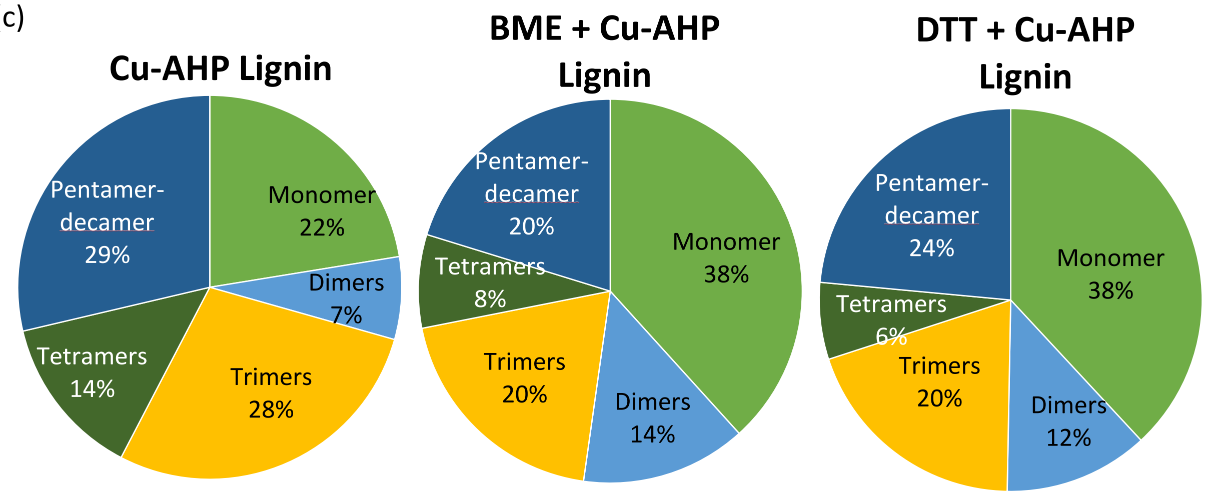

Figure S16. Analysis of Cu-AHP lignin depolymerization whole polymer and fragments. Cu-AHP lignin reactions were run with neat thiol and K_2CO_3 at 100 °C for 24 h. and worked up with water at pH 9. (a) GPC chromatogram of Cu-AHP lignin (grey), DTT (dithiothreitol)-treated Cu-AHP lignin (orange), and BME (2-mercaptoethanol)-treated Cu-AHP lignin (blue). The lignin polymer is highlighted from retention volume 5-10 mL while the lignin fragments smaller than 1,000 g/mol are highlighted from retention volume 10-30 mL. After 1,000 g/mol, molecules are separated by polarity with the disulfide products seen at ~13 mL. (b) Calculated molecular weights of M_n (number average molecular weight), M_w (weight average molecular weight), and M_p (high peak

molecular weight), and PDI (polydispersity index) from the top chromatogram of the lignin polymer. (c) Relative percentages of monomers, dimer, trimers, tetramers, and pentamers-decamers of the lignin fragments less than 1,000 g/mol for (a). Percentage is based on relative intensity of molecular weight ranges from 84-1068 g/mol. Percent polymers is broken down as follows: monomers 84-199 g/mol, dimers 200-299 g/mol, trimers 300-399 g/mol, tetramers 400-499 g/mol, and polymers 500-1068 g/mol. Values were obtained using a high-resolution LCMS-QTOF with all molecules with sulfur mass defects removed from analysis. GPC analysis shows a 60-73% molecular weight reduction in lignin. Soluble lignin fragment analysis suggests that there is significant cleavage by the thiol due to the larger % monomer compared to the lignin control.

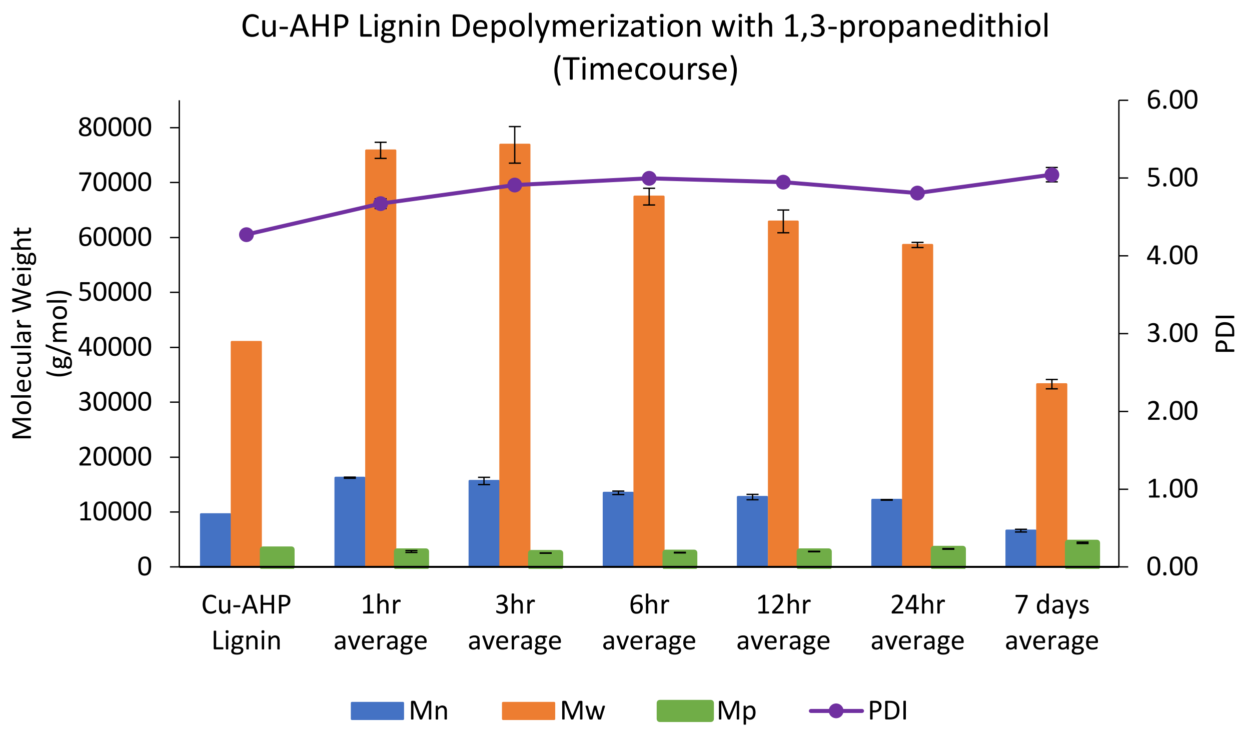

Figure S17. Susceptibility of Cu-AHP lignin depolymerization with 1,3-Propanedithiol over time. Cu-AHP lignin was reacted with neat 1,3-propanedithiol and K_2CO_3 at 100 °C for 1, 3, 6, 12, and 24 h. as well as 7 days. Calculated molecular weight M_n in blue (number average molecular weight), M_w in orange (weight average molecular weight), M_p in green (molecular weight at the high point on the polymer), and PDI in purple (polydispersity) was found for each time and compared to non-thiol treated Cu-AHP lignin. While 1,3-propanedithiol is very effective at cleaving dimers and synthetic polymers at high concentrations, this specific thiol appears slower in cleaving real lignin.

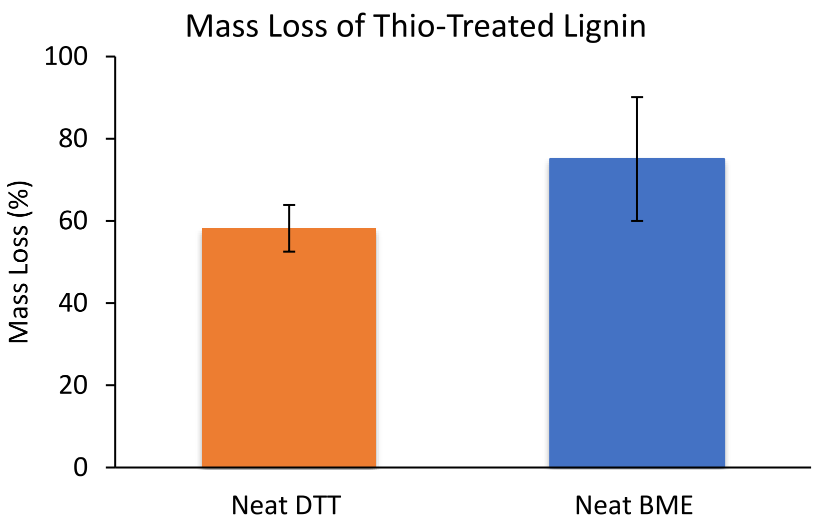

Figure S18. Determination of percent lignin cleavage by measuring mass loss after thiol-treatment. Mass loss (measured in triplicate) of Cu-AHP lignin precipitated using H_2SO_4 to pH 2 following reaction with either neat dithiothreitol (DTT) in orange or neat 2-mercaptoethanol (BME) in blue in stirring K_2CO_3 at 100 °C for 24 h. Control precipitation reactions for this work-up method are shown in Figure S24, demonstrating that without thiol mediated depolymerization, all Cu-AHP lignin mass is retained. This mass loss of 60-80% is presumably due to the formation of small fragments that are no longer insoluble at low pH demonstrating the success of thiol-mediated cleavage of lignin.

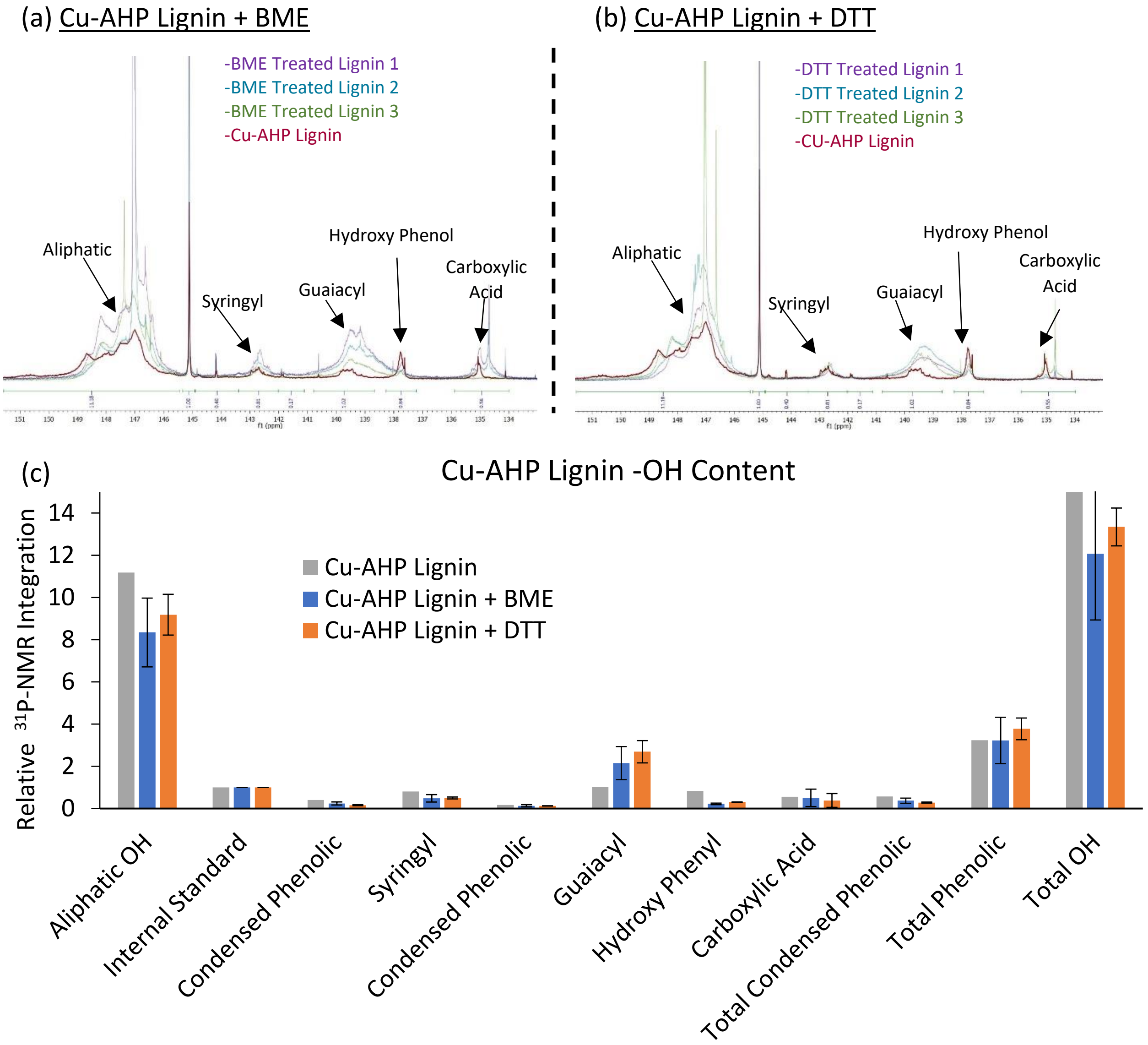

Figure S19. Measurement of -OH content to determine lignin cleavage. ^{31}P-NMR demonstrates relative concentration of hydroxy (-OH) groups via tagging. Changes in -OH content in lignin that has been depolymerized would suggest ether bond cleavage and -OH formation. Top: ^{31}P-NMR chromatograms of Cu-AHP lignin (red) and (a) Cu-AHP lignin reacted with neat BME (2-mercaptoethanol) and K_2CO_3 at 100° C in triplicate (purple, blue, and green) and (b) Cu-AHP lignin reacted with DTT (dithiothreitol) and K_2CO_3 at 100° C in triplicate (purple, blue, and green). Bottom: (c) Calculated values of -OH content from the spectra (top) integrated to an internal standard (cyclohexanol) with Cu-AHP lignin in grey, BME-treated Cu-AHP lignin in blue, and DTT-treated Cu-AHP lignin in orange. A significant difference between the guaiacyl -OH content of lignin and thiol treated lignin is observed suggesting that the guaiacyl phenolic monomer is readily cleaved through thiol-mediated lignin depolymerization.

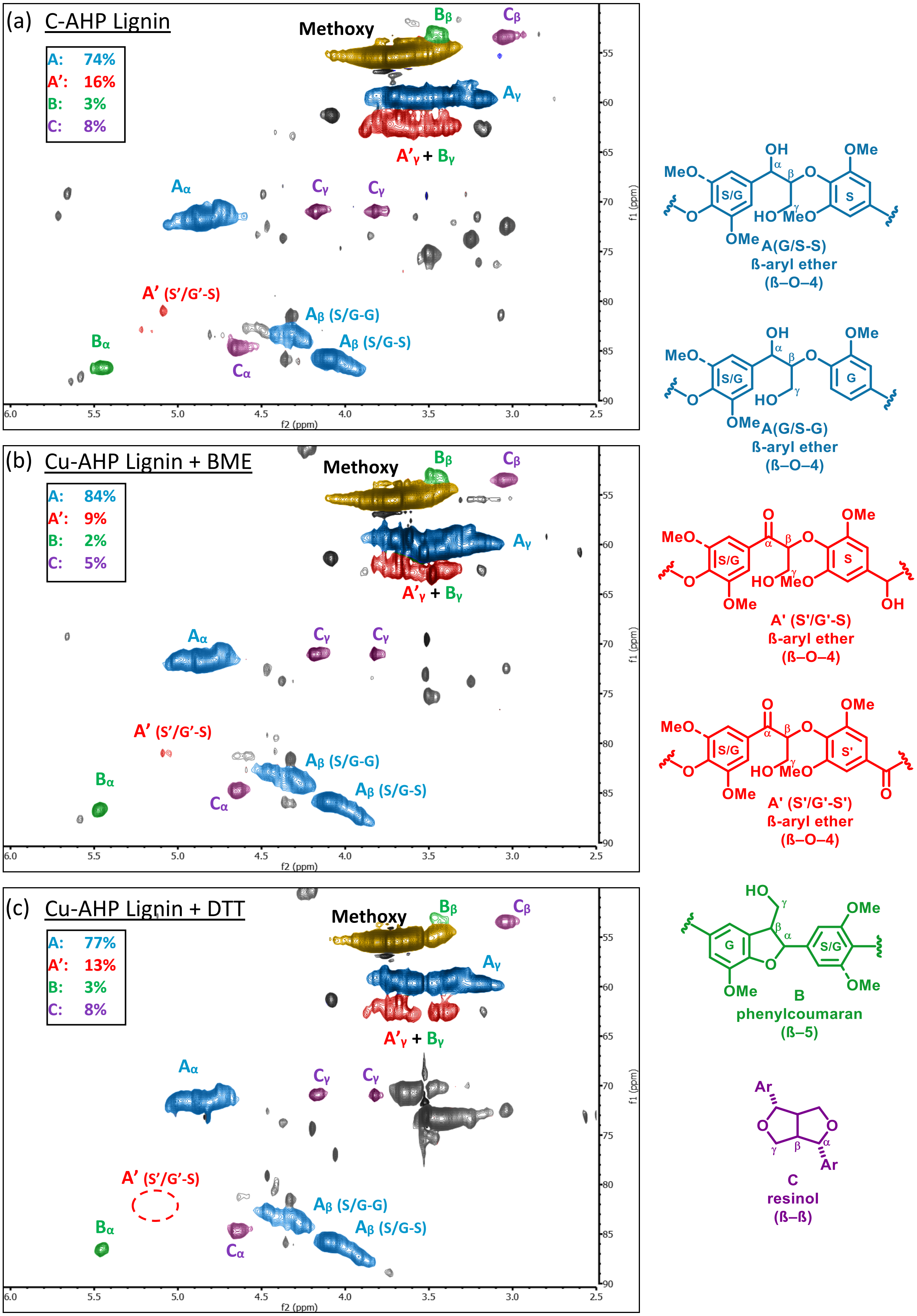
(a) C-AHP Lignin
A: 74%
A': 16%
B: 3%
C: 8%
Methoxy
Bβ
Cβ
Aγ
A'γ + Bγ
Aα
Cγ
Cγ
A' (S'/G'-S)
Aβ (S/G-G)
Aβ (S/G-S)
Bα
Cα
(b) Cu-AHP Lignin + BME
A: 84%
A': 9%
B: 2%
C: 5%
(c) Cu-AHP Lignin + DTT
A: 77%
A': 13%
B: 3%
C: 8%
f2 (ppm)
f1 (ppm)
A(G/S-S)
ß-aryl ether
(ß–O–4)
A(G/S-G)
ß-aryl ether
(ß–O–4)
A' (S'/G'-S)
ß-aryl ether
(ß–O–4)
A' (S'/G'-S')
ß-aryl ether
(ß–O–4)
B
phenylcoumaran
(ß–5)
C
resinol
(ß–ß)

Figure S20. HSQC NMR of Cu-AHP lignin demonstrating changes to the polymer structure after depolymerization with thiol. Cu-AHP lignin was reacted with neat BME (2-mercaptoethanol) or neat DTT (dithiothreitol) with stirring K_2CO_3 in 100 °C for 24 h. The remaining polymer was precipitated and worked up according to Section II. (a) Cu-AHP lignin, (b) BME treated Cu-AHP lignin, and (c) DTT treated Cu-AHP lignin. The relative volume integrals of characteristic peaks are given. Cross peaks are color coded according each linkage assigned. Cu-AHP lignin is slightly oxidized as shown in (a) by peaks A' (red). A small amount of cleavage evidence is seen after thiol-treatment of lignin (b-c) seen from the decrease in A' integration (red). The oxidized β-O-4 linkage, assigned to the red cross peaks, is the only expected integrals to decrease by thiol depolymerization due to the carbonyl requirement to activate nucleophilic cleavage.

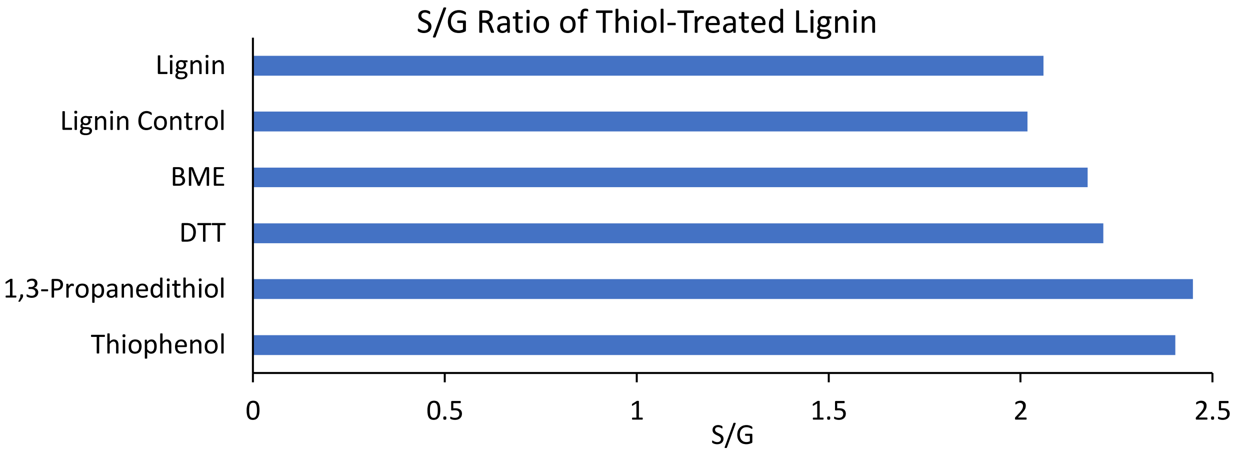

Figure S21. Thioacidolysis S/G ratio of monomer units in remaining lignin polymer after thiol-treatment. Cu-AHP lignin was subjected to neat thiol: BME (2-mercaptoethanol), DTT (dithiothreitol), 1,3-propanedithiol, or thiophenol and K_2CO_3 at 100 °C for 24 h. The mixture was worked up with water, pH adjusted to 2 with H_2SO_4, washed, frozen, lyophilized, and submitted for thioacidolysis analysis. *Y*-axis: lignin is Cu-AHP lignin powder, lignin control is Cu-AHP lignin worked up in the same manner as thiol-treated lignin, BME is BME-treated Cu-AHP lignin, DTT is DTT-treated Cu-AHP lignin, 1,3-propanedithiol is 1,3-propanedithiol-treated Cu-AHP lignin, and thiophenol is thiophenol-treated Cu-AHP lignin. The little change in S/G ratio indicates no preference in lignin unit cleavage for syringyl versus guaiacyl monomers. Very little difference is seen between lignin and lignin control suggesting that the work-up does not change the thioacidolysis results.

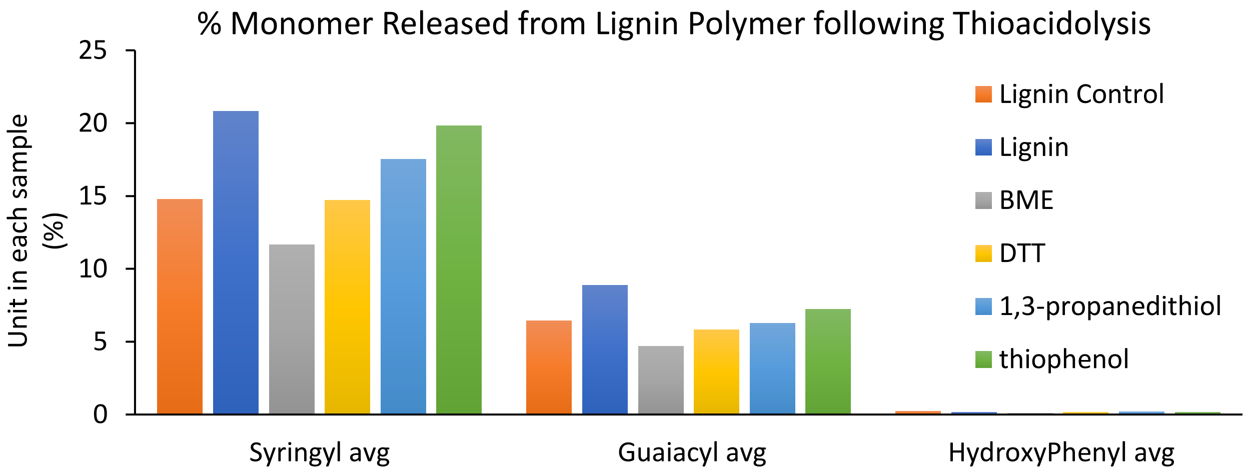

Figure S22. Thioacidolysis % monomer in thiol-treated and non-treated lignin polymer. Cu-AHP lignin was subjected to neat thiol: BME (2-mercaptoethanol), DTT (dithiothreitol), 1,3-propanedithiol, or thiophenol and K_2CO_3 at 100 °C for 24 h. The mixture was worked up with water, pH adjusted to 2 with H_2SO_4, washed, frozen, lyophilized, and submitted for thioacidolysis analysis. Labels are as follows: lignin is Cu-AHP lignin powder, lignin control is Cu-AHP lignin worked up in the same manner as thiol-treated lignin, BME is BME-treated Cu-AHP lignin, DTT is DTT-treated Cu-AHP lignin, 1,3-propanedithiol is 1,3-propanedithiol-treated Cu-AHP lignin, and thiophenol is thiophenol-treated Cu-AHP lignin. The results indicate that (BME)- and (DTT)-treated lignin have decreased thioacidolysis monomer yields, potentially indicating that those monomers may have already been released during thiol-mediated depolymerization. This would be due to less β-O-4 linkages remaining to react during the analysis. No hydroxy phenol units were seen because the Cu-AHP lignin used was made from poplar which has a low H-content.

XI. Cleavage of Oxidized Lignin

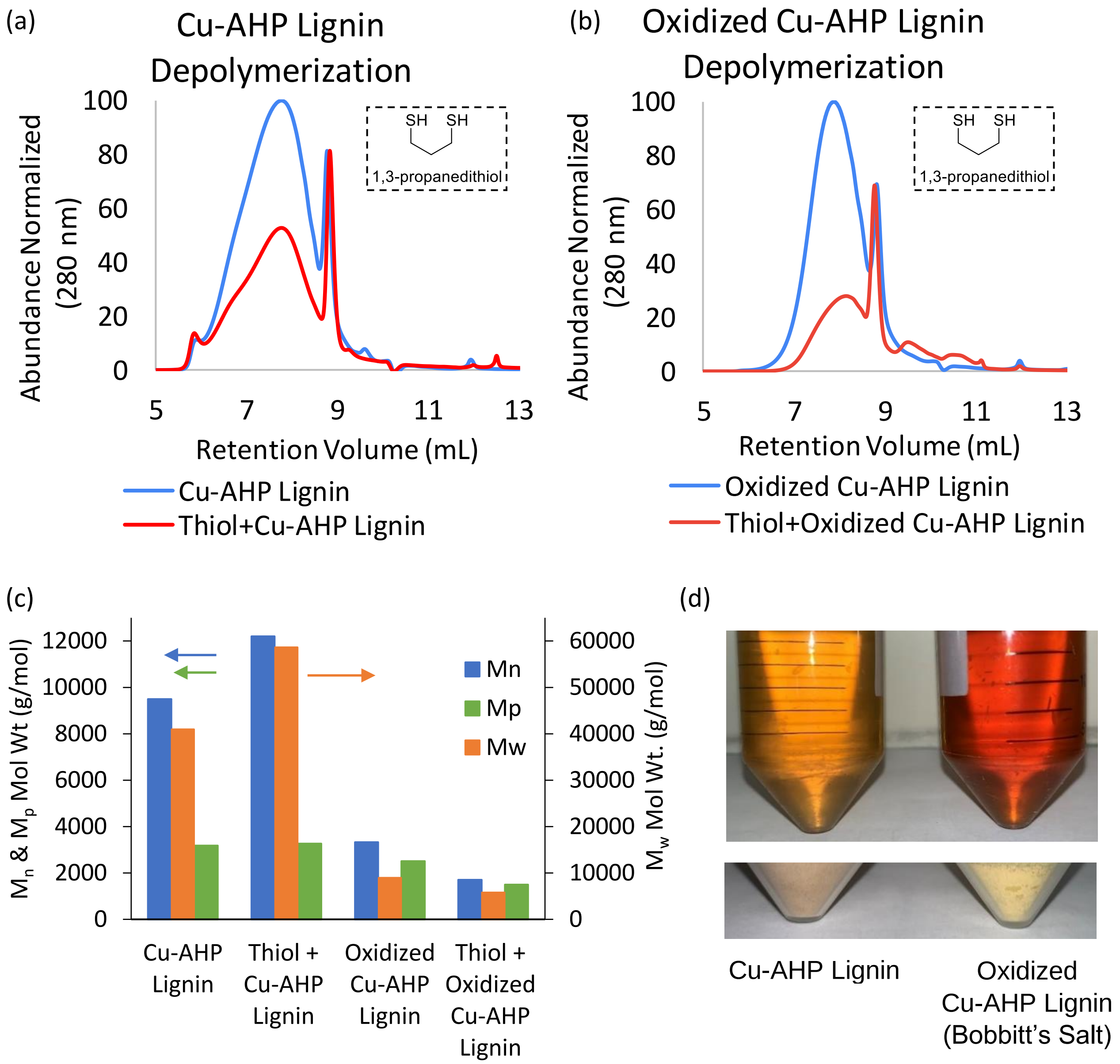

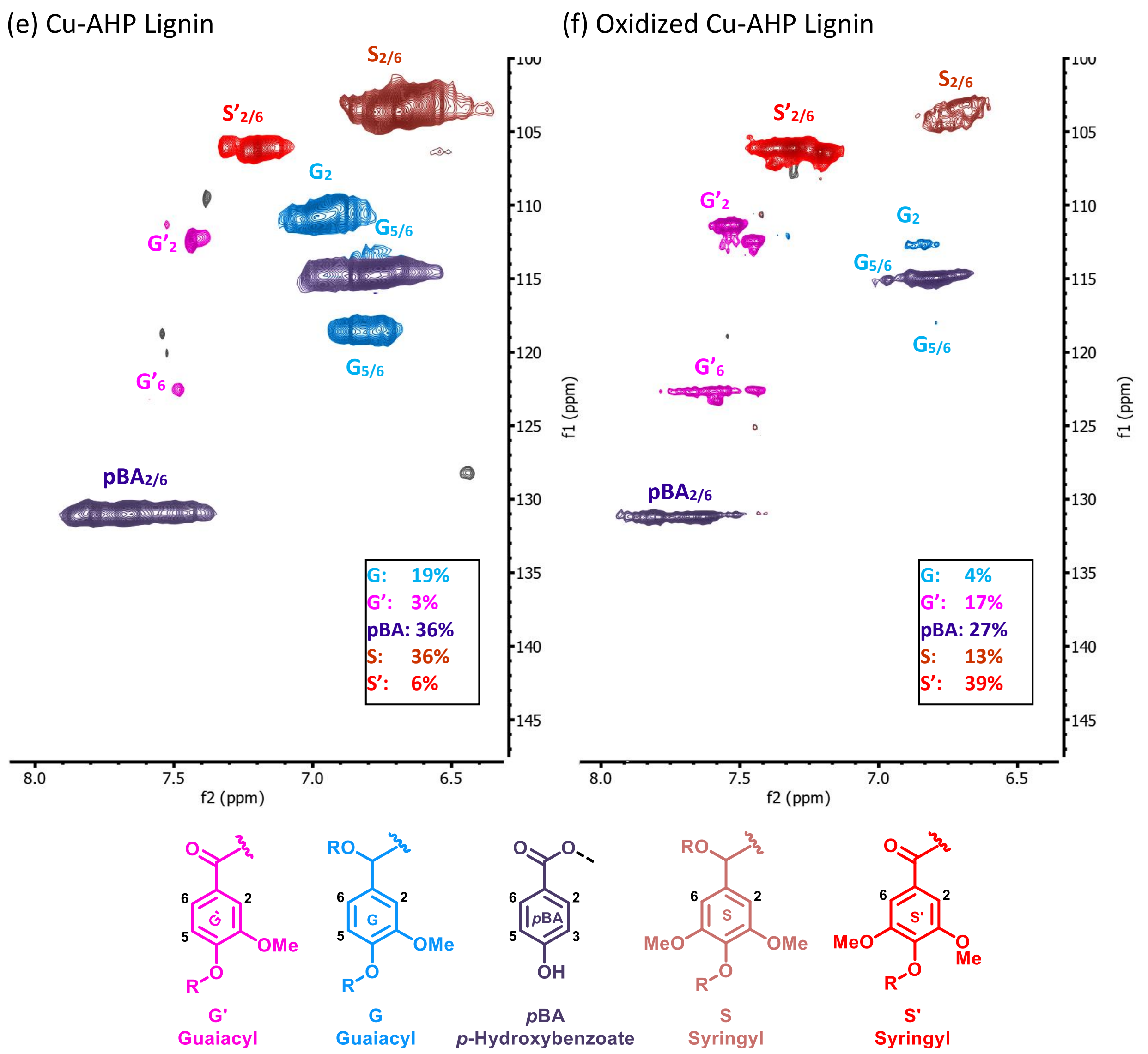

Figure S23. Comparison of Cu-AHP lignin depolymerization on oxidized vs non-oxidized lignin. Cu-AHP lignin was reacted with neat 1,3-propanedithiol and stirring K_2CO_3 in 100 °C for 24 h. (a) GPC chromatogram of Cu-AHP lignin (blue) and thiol treated Cu-AHP lignin (red). (b) GPC chromatogram of Cu-AHP lignin oxidized with Bobbitt's Salt[14] (blue) and thiol treated oxidized Cu-AHP lignin (red). Visually the oxidized lignin appears to undergo a larger decrease in molecular weight than the non-oxidized lignin. (c) Calculated molecular weights M_n in blue (number average molecular weight), M_w in orange (weight average molecular weight), and M_p in green (molecular weight at the high point on the polymer) for (a) and (b) with the M_n and M_p axis on the left and the M_w axis on the right. The oxidized lignin has a larger decrease in molecular weight when subjected to thiol than the unoxidized lignin. After depolymerization oxidized Cu-AHP lignin's M_n, M_w, and M_p decreased by 49%, 36%, and 40%, respectively. (d) Both unoxidized

(left) and oxidized (right) Cu-AHP lignin is completely soluble in alkaline water. The bottom picture shows the dry lignin powders' color before the reaction: the oxidized lignin is a light yellow and the unoxidized lignin is a tan color. (e-f) HSQC-NMR of (e) Cu-AHP lignin and (f) oxidized Cu-AHP lignin from Bobbitt's salt oxidation. The relative volume integrals of characteristic peaks are given. Cross peaks are color coded according each linkage assigned. The Cu-AHP lignin exhibits about 14% oxidation of both S and G units due to the oxidative extraction process. After oxidation of the Cu-AHP lignin with Bobbitt's salt, the S and G units exhibit approximately 74% and 81% oxidation levels, respectively. From these results, it is clear that oxidized lignin is highly susceptible to thiol-mediated depolymerization. The sharp peak near 9 mL retention volume in the GPC absorbs at 280 nm and is assumed to be an aromatic oligomer. Based on the polysulfonated-styrene molecular weight standards, this peak would be assigned a molecular weight of <1000 g/mol, but it is important to recognize that these standards may not ideally represent the behavior of phenylpropanoid oligomers.

XII. Lignin Control Experiments

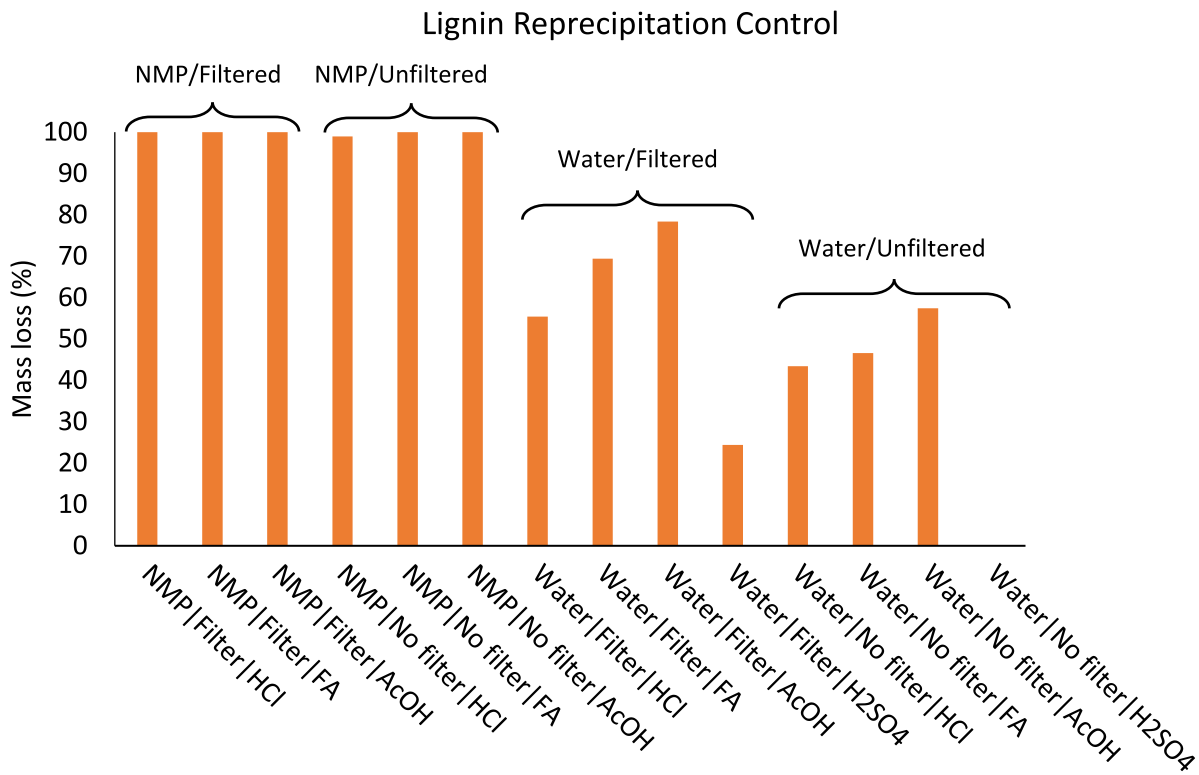

Figure S24. Control experiment to test lignin work-up precipitation to obtain accurate mass loss yields. Cu-AHP lignin was solubilized in NMP or water and K_2CO_3, filtered or not filtered, and then precipitated with different acids (hydrochloric acid (HCl), formic acid (FA), acidic acid (AcOH), or sulfuric acid (H_2SO_4)) to determine the recoverability of the lignin during a work-up procedure to obtain accurate mass loss yields. Sulfuric acid precipitation in water with no filtering gave quantitative recovery of lignin (0% mass loss) as indicated by no bar shown for mass loss.

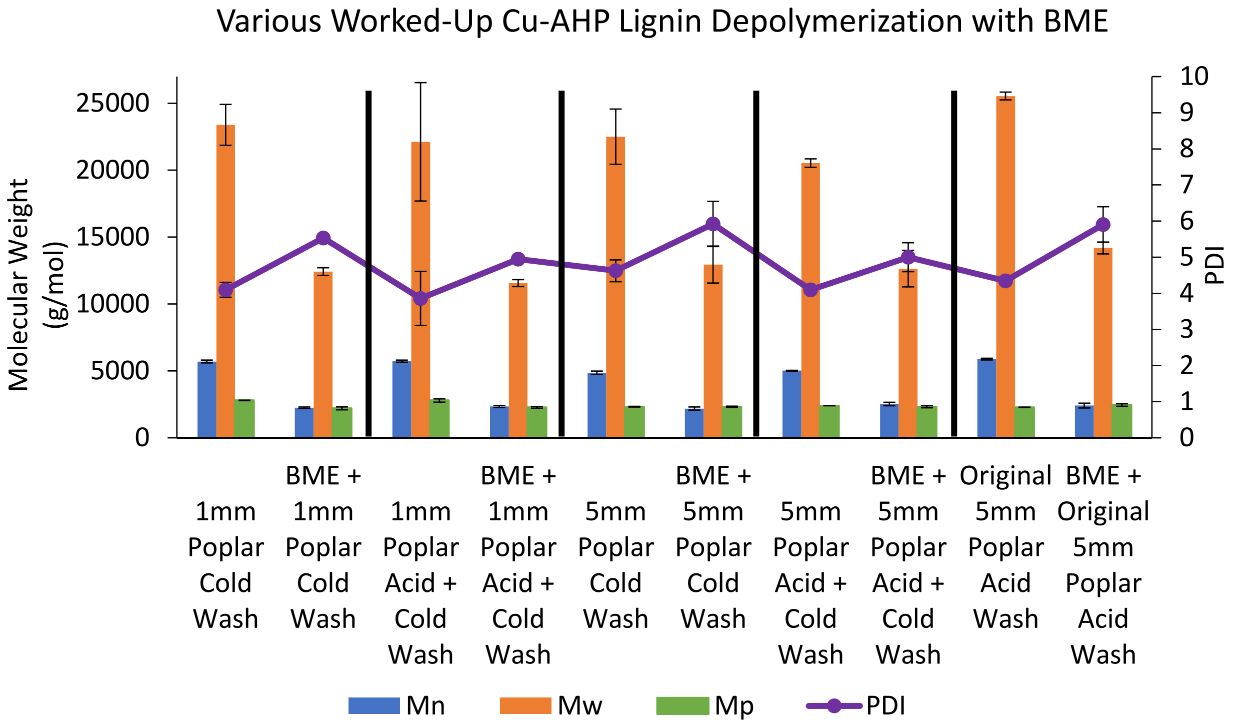

Figure S25. Control reactions to determine changes in molecular weight based on differences in Cu-AHP lignin extraction procedure: grind size of wood and work-up procedure of extracting the acid insoluble lignin. Cu-AHP lignin was made with either 1mM or 5mm wood grind size and combined with different precipitation procedures to extract the lignin: wash with cold water 3x, wash with acid 2x and cold water 1x, or the usual procedure of wash with acidic water 3x (labeled as original). These different Cu-AHP lignin's were then subjected to depolymerization with neat BME (2-mecaptoethanol) and K_2CO_3 at 100 °C for 24 h. and compared with the original procedure mentioned in Section II: Lignin Extraction Protocol. Calculated molecular weights M_n in blue (number average molecular weight), M_w in orange (weight average molecular weight), M_p in green (molecular weight at the high point on the polymer), and PDI in purple (polydispersity) between the samples indicate that wood mesh size and work-up do not effect depolymerization results. Samples were run in triplicate.

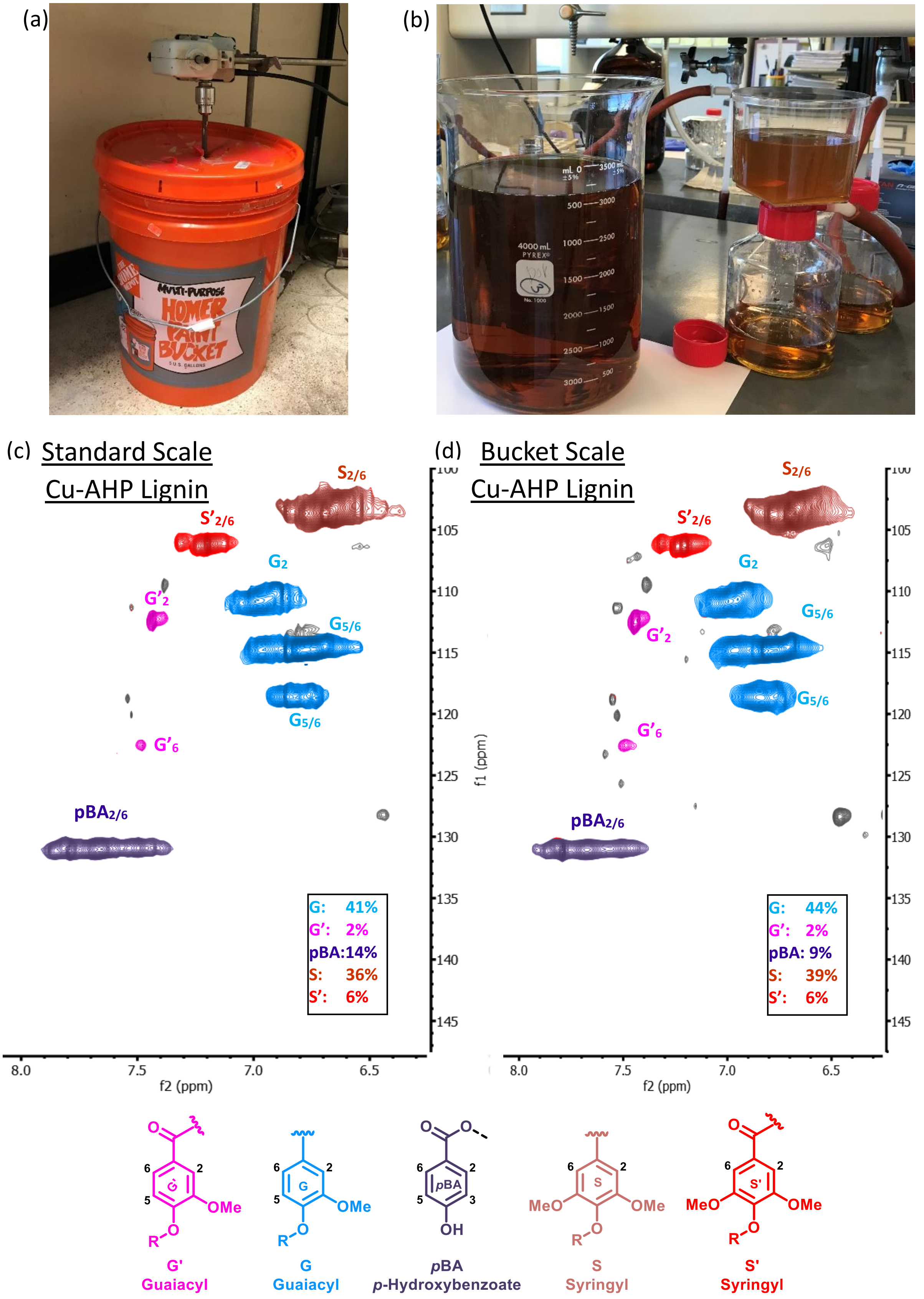
(a)
(b)
(c) Standard Scale
Cu-AHP Lignin
(d) Bucket Scale
Cu-AHP Lignin
S2/6
S'2/6
G2
G'2
G5/6
G'6
pBA2/6
G: 41%
G': 2%
pBA:14%
S: 36%
S': 6%
G: 44%
G': 2%
pBA: 9%
S: 39%
S': 6%
f1 (ppm)
f2 (ppm)
OMe
MeO
OH
R
G'
Guaiacyl
G
Guaiacyl
pBA
p-Hydroxybenzoate
S
Syringyl
S'
Syringyl

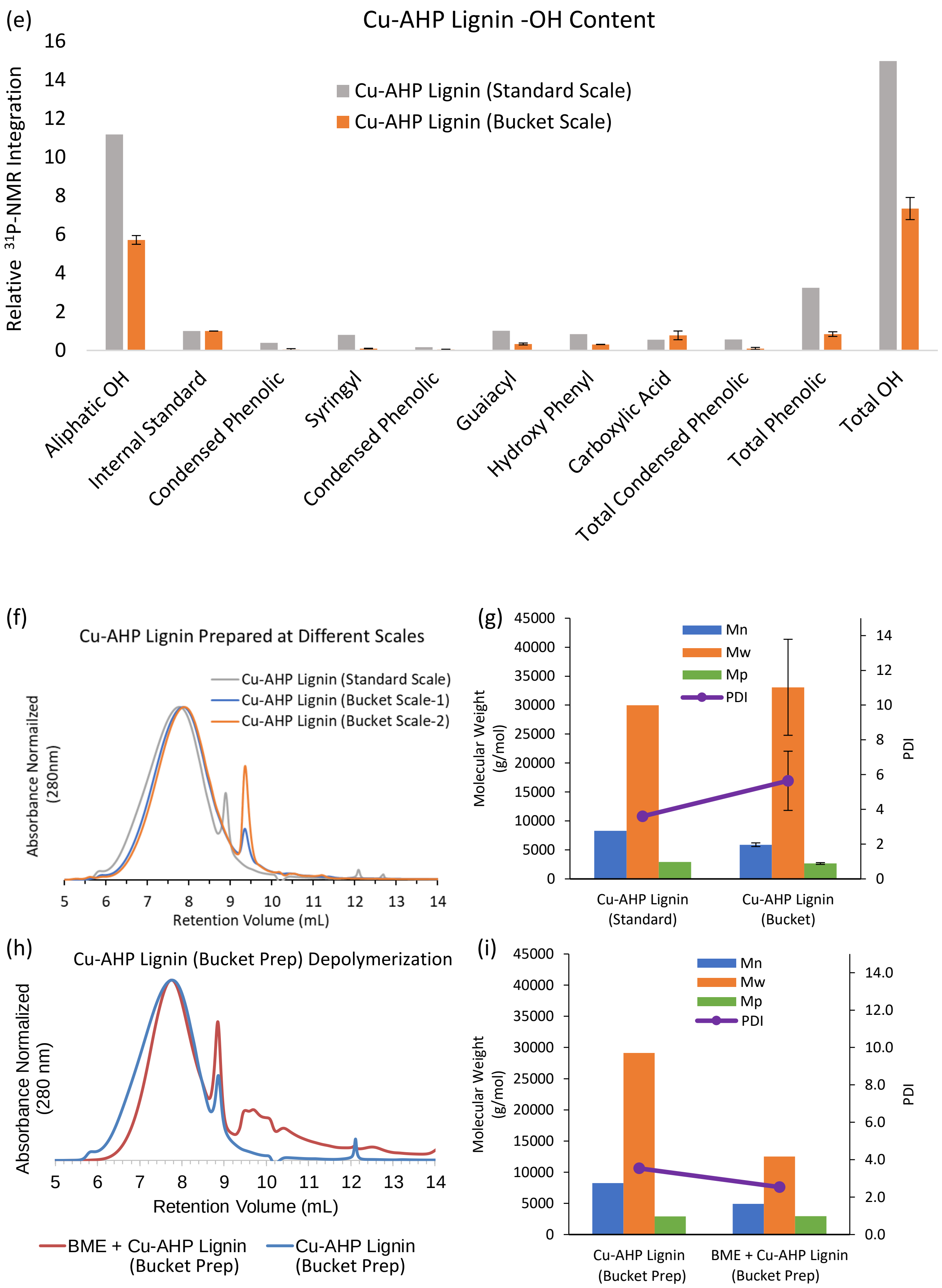
(e)
Cu-AHP Lignin -OH Content
Relative 31P-NMR Integration
16
14
12
10
8
6
4
2
0
Cu-AHP Lignin (Standard Scale)
Cu-AHP Lignin (Bucket Scale)
Aliphatic OH
Internal Standard
Condensed Phenolic
Syringyl
Condensed Phenolic
Guaiacyl
Hydroxy Phenyl
Carboxylic Acid
Total Condensed Phenolic
Total Phenolic
Total OH
(f)
Cu-AHP Lignin Prepared at Different Scales
Cu-AHP Lignin (Standard Scale)
Cu-AHP Lignin (Bucket Scale-1)
Cu-AHP Lignin (Bucket Scale-2)
Absorbance Normailzed (280nm)
5
6
7
8
9
10
11
12
13
14
Retention Volume (mL)
(g)
Molecular Weight (g/mol)
45000
40000
35000
30000
25000
20000
15000
10000
5000
0
Mn
Mw
Mp
PDI
14
12
10
8
6
4
2
0
PDI
Cu-AHP Lignin (Standard)
Cu-AHP Lignin (Bucket)
(h)
Cu-AHP Lignin (Bucket Prep) Depolymerization
Absorbance Normalized (280 nm)
5
6
7
8
9
10
11
12
13
14
Retention Volume (mL)
BME + Cu-AHP Lignin (Bucket Prep)
Cu-AHP Lignin (Bucket Prep)
(i)
Molecular Weight (g/mol)
45000
40000
35000
30000
25000
20000
15000
10000
5000
0
Mn
Mw
Mp
PDI
14.0
12.0
10.0
8.0
6.0
4.0
2.0
0.0
PDI
Cu-AHP Lignin (Bucket Prep)
BME + Cu-AHP Lignin (Bucket Prep)

Figure S26. Control study to determine if scale up of Cu-AHP lignin extraction affects its thiol-mediated depolymerization. (a) Cu-AHP reaction in a 5-gallon bucket which will produce a lignin stream. (b) Lignin liquor from bucket scale Cu-AHP pretreatment before acid precipitation. (c-d) HSQC 2D-NMR of (c) Cu-AHP lignin made at standard scale, (d) Cu-AHP lignin made at bucket scale. The relative volume integrals of characteristic peaks are given. Cross peaks are color coded according each linkage assigned. (e) -OH content calculated from ^{31}P-NMR of Cu-AHP lignin made at standard scale and duplicates made at bucket scale. (f) GPC chromatogram comparison of Cu-AHP lignin made at standard scale and bucket scale in duplicate. (g) Calculated molecular weight distributions of M_n in blue (number average molecular weight), M_w in orange (weight average molecular weight), M_p in green (molecular weight at the high point on the polymer), and PDI in purple (polydispersity) of (f). (h) GPC chromatogram of Cu-AHP lignin (bucket scale) compared to the BME (2-mercaptoethanol) treated Cu-AHP lignin (bucket scale) reacted in neat conditions at 100 °C for 24 h. (i) Calculated molecular weight distributions of M_n in blue, M_w in orange, M_p in green, and PDI in purple of (h). Lignin prepared in a large bucket scale had similar molecular weights as the standard scale Cu-AHP lignin but had less phenolic and aliphatic -OH's which may be due to increases in oxidation. NMR-analysis determined very little structural changes between standard and bucket scale Cu-AHP lignin. The scaled-up Cu-AHP lignin was depolymerized using BME with decreases in M_n, M_w, and PDI similar to results seen with standard-scale Cu-AHP lignin.

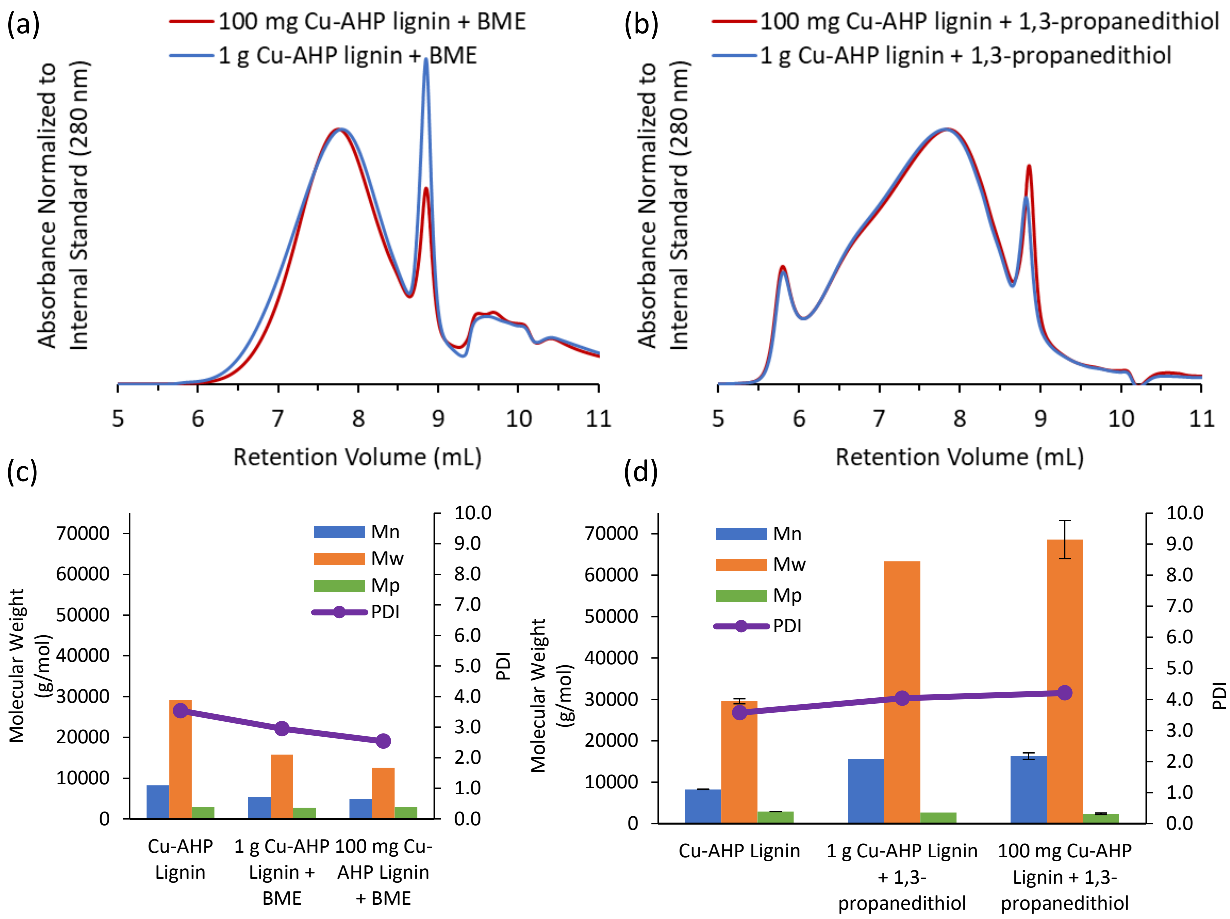

Figure S27. Control experiment to determine the scalability of thiol-mediated lignin depolymerization. Cu-AHP lignin reacted with neat BME (2-mercaptoethanol) or neat 1,3-propanedithiol and K_2CO_3 at 100 °C for 24 h. (a) GPC chromatogram of BME-treated Cu-AHP lignin at a 1-gram scale (blue) and a 100-milligram scale (red). (b) GPC chromatogram of 1,3-propanedithiol-treated Cu-AHP lignin at a 1-gram scale (blue) and a 100-milligram scale (red). Calculated molecular weights M_n in blue (number average molecular weight), M_w in orange (weight average molecular weight), M_p in green (molecular weight at the high point on the polymer: seen at 7.75 mL above), and PDI in purple (polydispersity) of Cu-AHP lignin compared to (c) the 1-g and 100-mg scale of BME-treated lignin and (d) the 1-g and 100-mg scale of the 1,3-propanedithiol treated lignin. From these results it can be stated that the thiol depolymerization strategy is scalable from 100 mg to 1 g.

XIII. Proposed Electrochemical Cycle for the Thiol-Mediated Oxidized β-O-4 Cleavage

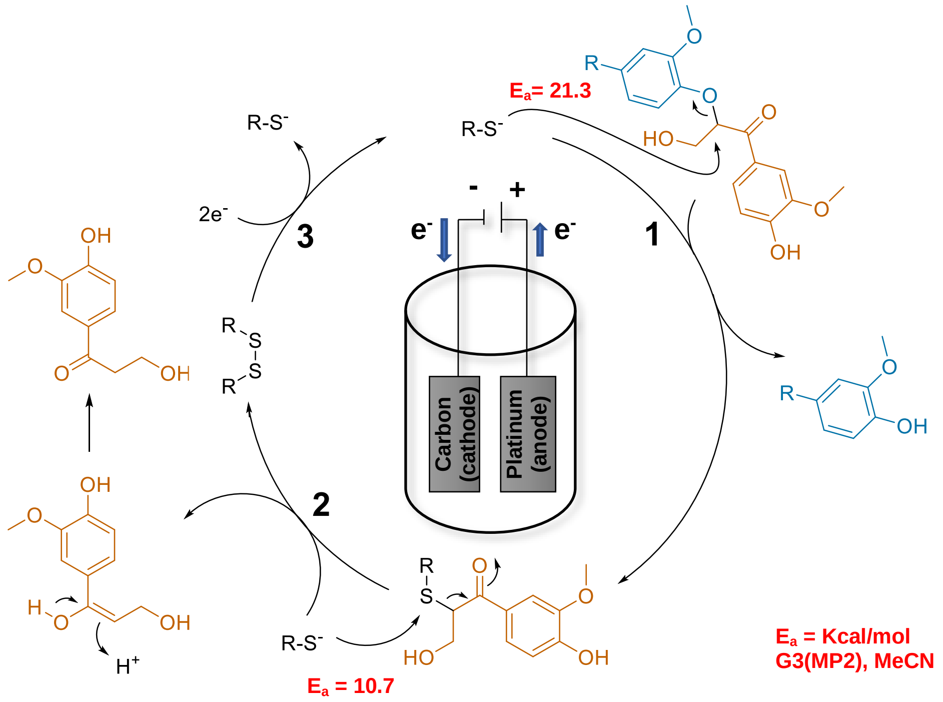

Figure S28. Organocatalytic cycle envisioned for the thiol-mediated nucleophilic cleavage of β-aryl ether bonds followed by a 2e⁻ reduction to recycle the mediator. Step 1: Nucleophilic organic thiol attack on an oxidized β-aryl ether bond of lignin displaces a phenolic unit. Calculated activation energy in MeCN: 21.3 Kcal/mol.[11] Step 2: A second thiol attacks the thioether intermediate, forming a disulfide, and releasing the aryl propanone fragment. Calculated activation energy in MeCN: 10.7 Kcal/mol. Step 3: The thiol can be regenerated via a 2-electron electrochemical reduction of the disulfide bond, potentially enabling a net electrocatalytic lignin cleavage process. Electrochemical cell demonstrated inside the catalytic cycle.

XIV. References

[1] D. W. Ma, Q. Cai, *Organic Letters* **2003**, *5*, 3799-3802.

[2] A. Tlili, F. Monnier, M. Taillefer, *Chemistry-a European Journal* **2010**, *16*, 12299-12302.

[3] T. Hu, T. Schulz, C. Torborg, X. Chen, J. Wang, M. Beller, J. Huang, *Chemical Communications* **2009**, 7330-7332.

[4] S. Murthy, J. Desantis, P. Verheugd, M. M. Maksimainen, H. Venkannagari, S. Massari, Y. Ashok, E. Obaji, Y. Nkizinkinko, B. Luescher, O. Tabarrini, L. Lehtio, *European Journal of Medicinal Chemistry* **2018**, *156*, 93-102.

[5] M. Wang, L. H. Li, J. M. Lu, H. J. Li, X. C. Zhang, H. F. Liu, N. C. Luo, F. Wang, *Green Chemistry* **2017**, *19*, 702-706.

[6] R. Kuwano, H. Kusano, *Organic Letters* **2008**, *10*, 1979-1982.

[7] T. Kishimoto, Y. Uraki, M. Ubukata, *Organic & Biomolecular Chemistry* **2008**, *6*, 2982-2987; T. Kishimoto, Y. Uraki, M. Ubukata, *Organic & Biomolecular Chemistry* **2006**, *4*, 1343-1347; T. Kishimoto, Y. Uraki, M. Ubukata, *Organic & Biomolecular Chemistry* **2005**, *3*, 1067-1073.

[8] H. Kim, J. Ralph, *Organic & Biomolecular Chemistry* **2010**, *8*, 576-591; S. D. Mansfield, H. Kim, F. Lu, J. Ralph, *Nature Protocols* **2012**, *7*, 1579-1589.

[9] S. Kalami, M. Arefmanesh, E. Master, M. Nejad, *Journal of Applied Polymer Science* **2017**, *134*.

[10] A. E. Harman-Ware, C. Foster, R. M. Happs, C. Doeppke, K. Meunier, J. Gehan, F. Yue, F. Lu, M. F. Davis, *Biotechnology Journal* **2016**, *11*, 1268-1273.

[11] G. E. Klinger, Y. Zhou, P. Hao, J. Robbins, J. M. Aquilina, J. E. Jackson, E. L. Hegg, *Chemsuschem* **2019**, *12*, 4775-4779.

[12] D. L. Gall, J. Ralph, T. J. Donohue, D. R. Noguera, *Environ. Sci. Technol.* **2014**, *48*, 12454-12463.

[13] R. Rinaldi, R. Jastrzebski, M. T. Clough, J. Ralph, M. Kennema, P. C. A. Bruijnincx, B. M. Weckhuysen, *Angewandte Chemie-International Edition* **2016**, *55*, 8164-8215.

[14] A. Rahimi, A. Azarpira, H. Kim, J. Ralph, S. S. Stahl, *Journal of the American Chemical Society* **2013**, *135*, 6415-6418.

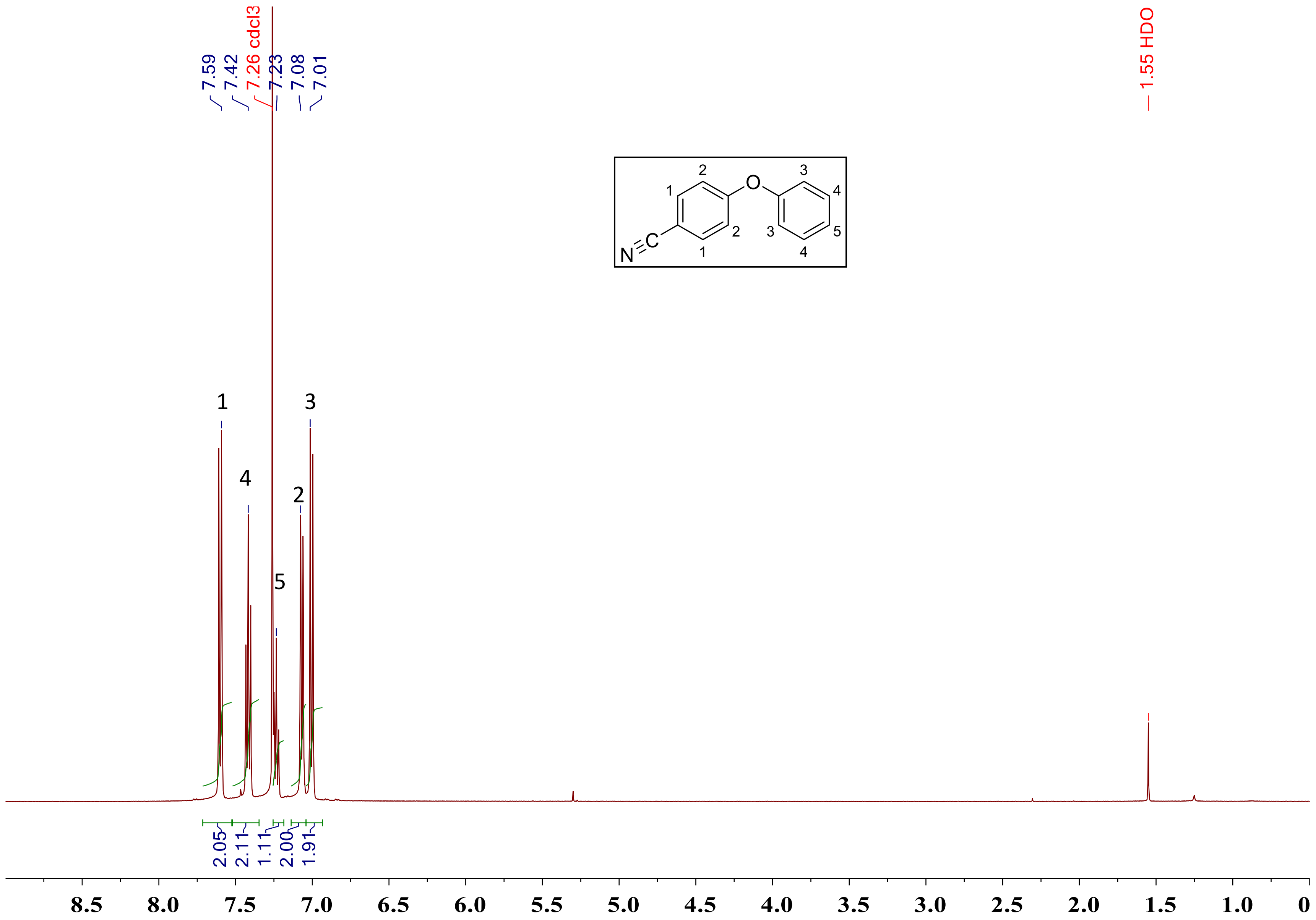

Figure S29. 4-Phenoxybenzonitrile

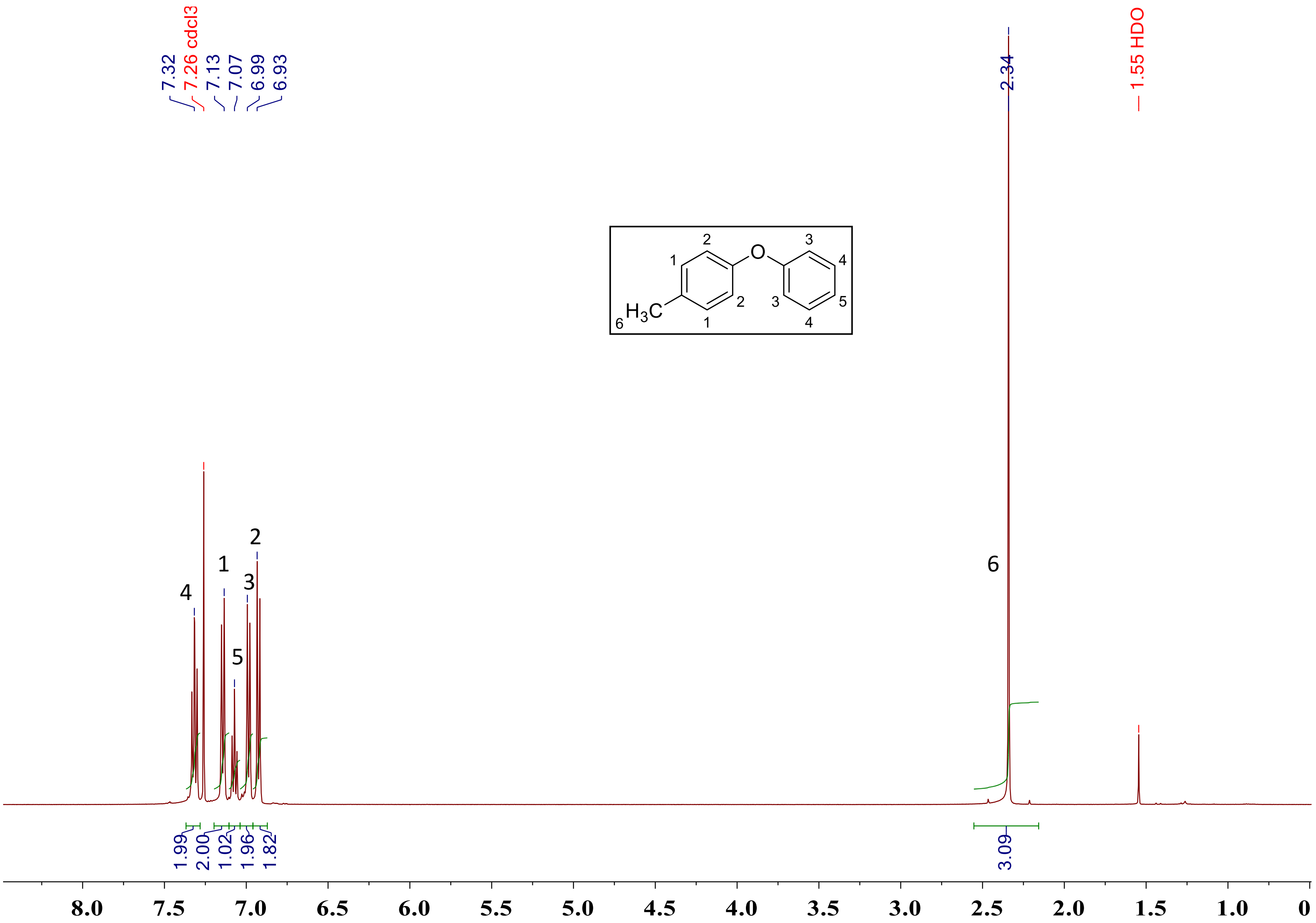

Figure S30. 4-Tolyl phenyl ether

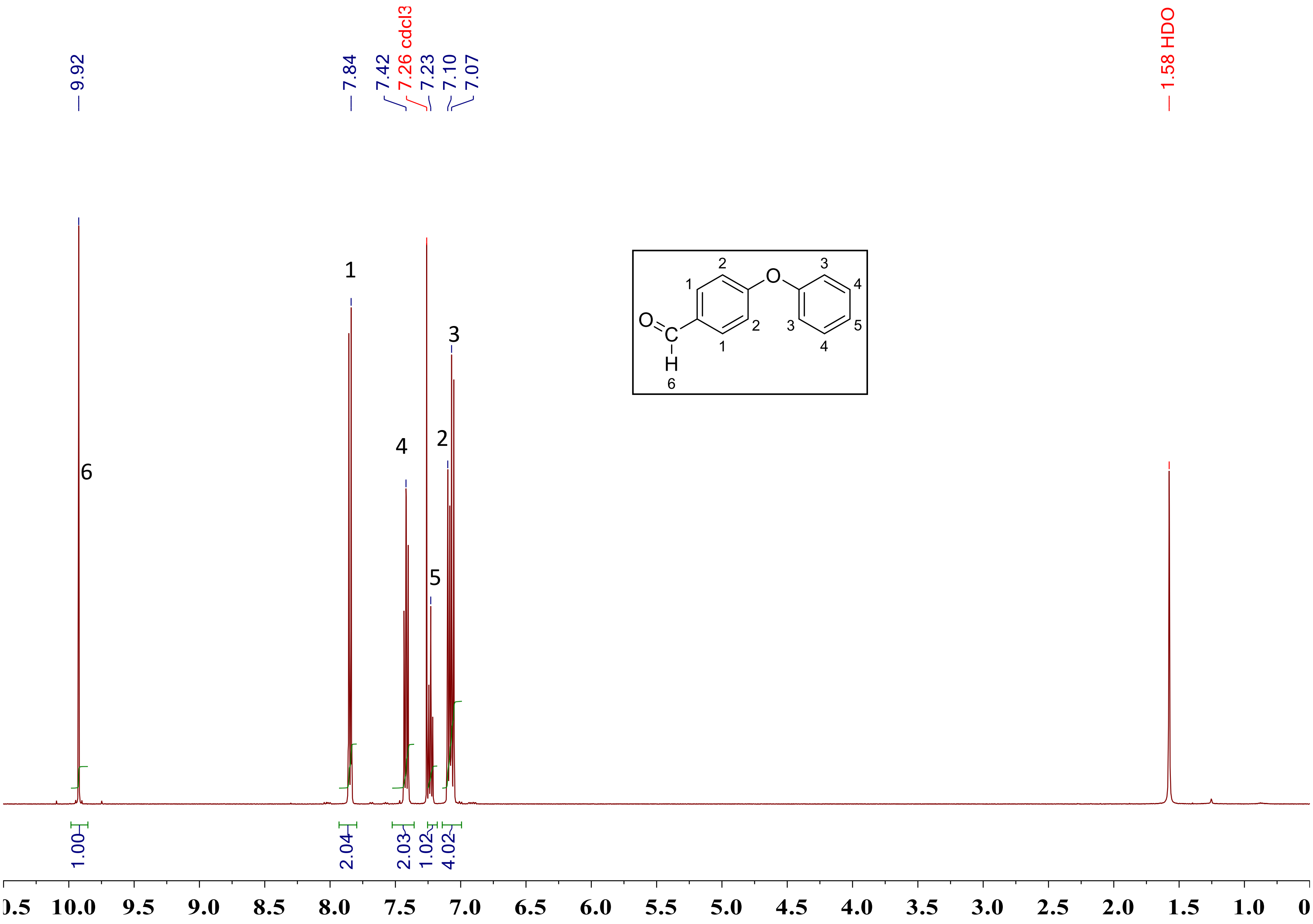

Figure S31. 4-Phenoxybenzaldehyde

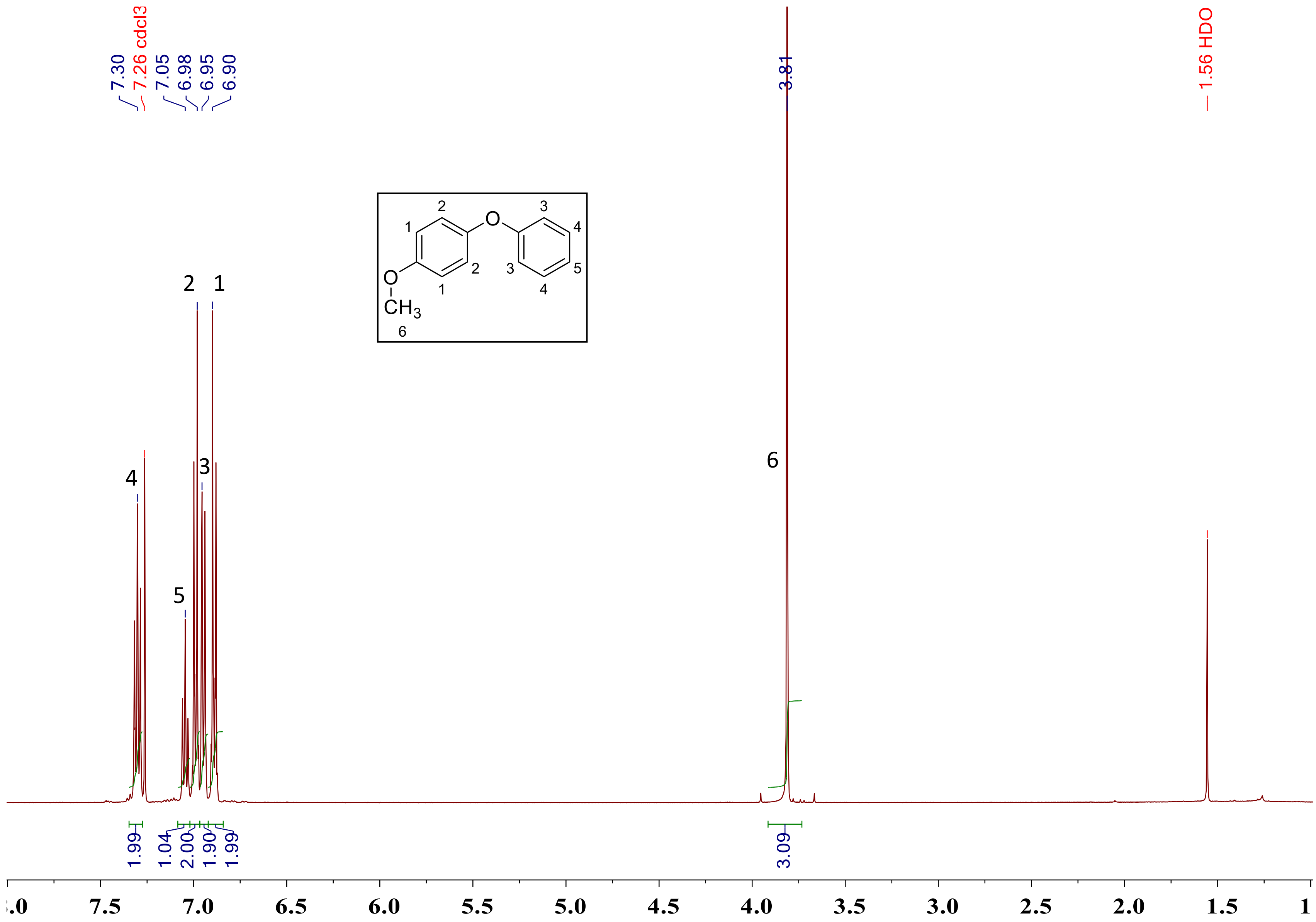

Figure S32. 1-Methoxy-4-phenoxybenzene

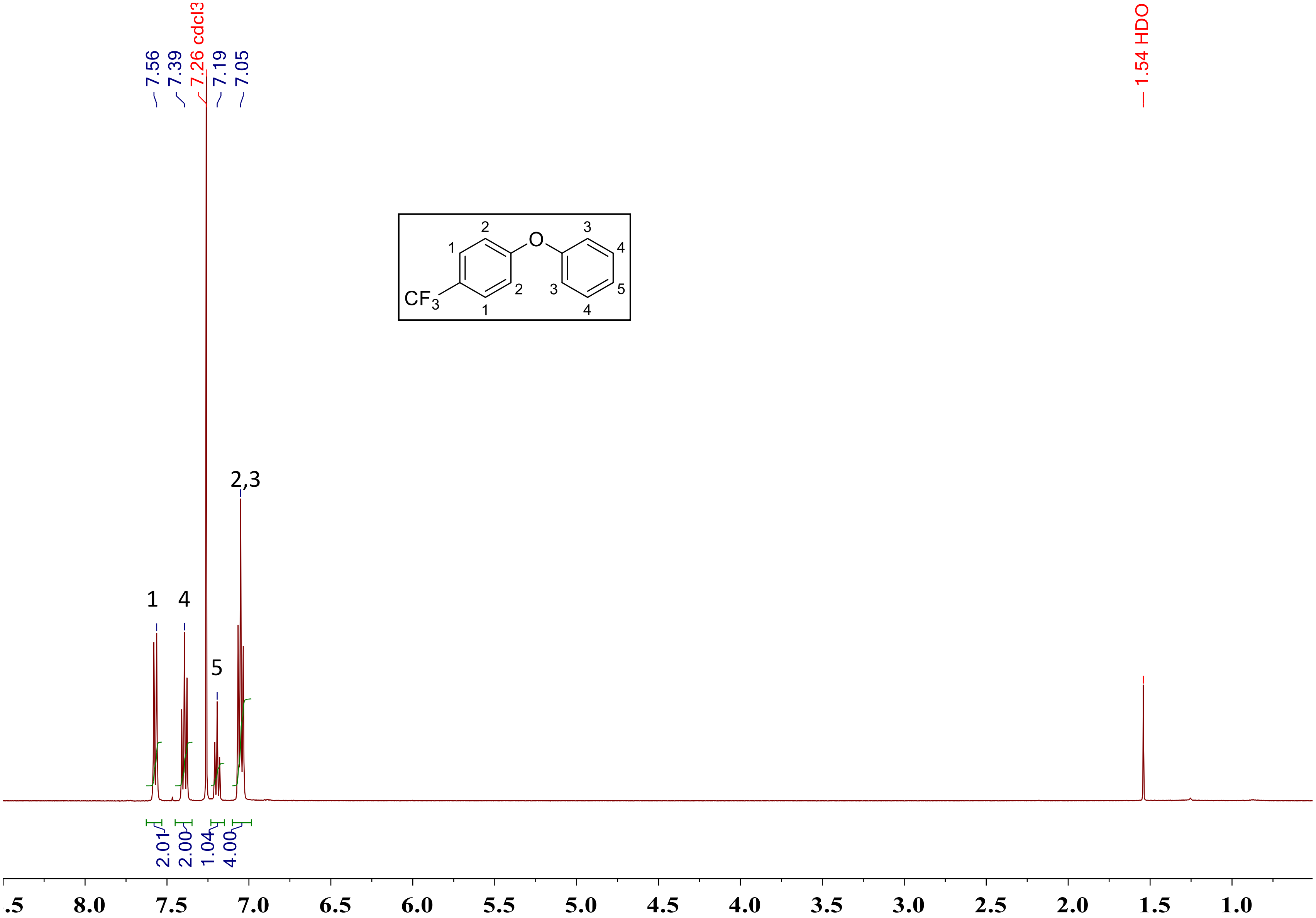

Figure S33. 1-Phenoxy-4-(trifluoromethyl)benzene

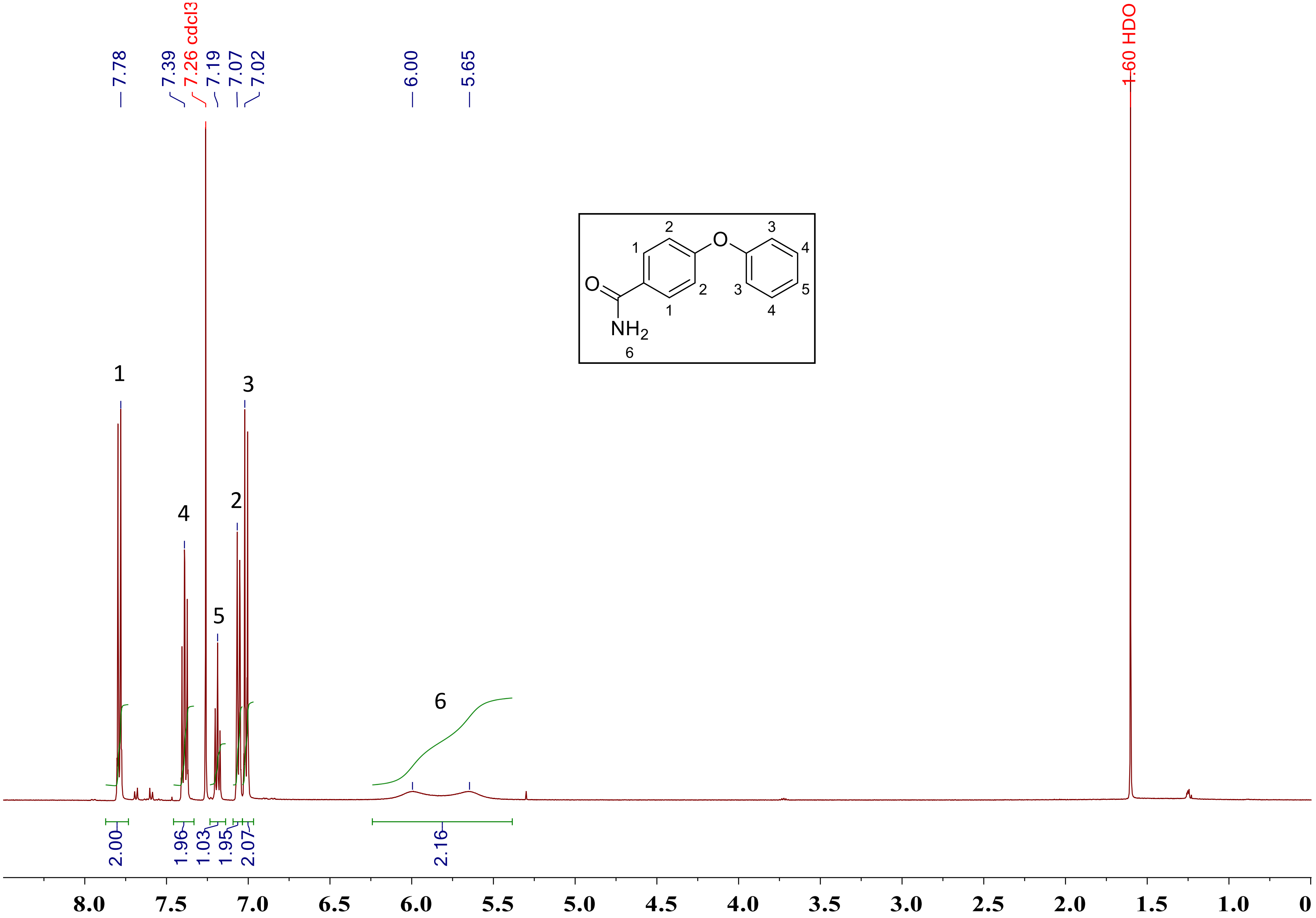

Figure S34. 4-phenoxybenzamide

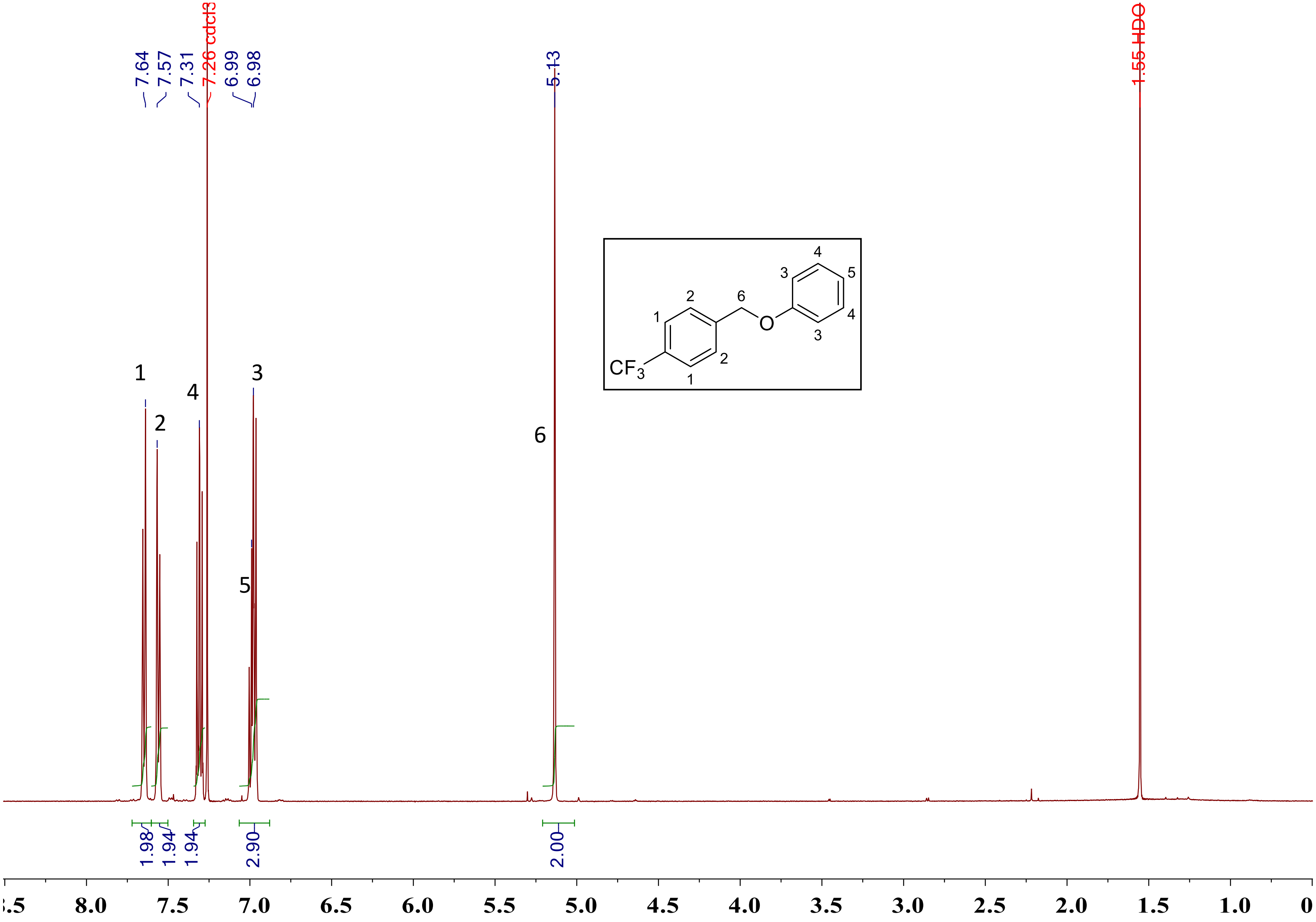

Figure S35. 1-(Phenoxymethyl)-4-(trifluoromethyl)benzene
